# Structural basis for translational control by the human 48S initiation complex

**DOI:** 10.1038/s41594-024-01378-4

**Published:** 2024-09-17

**Authors:** Valentyn Petrychenko, Sung-Hui Yi, David Liedtke, Bee-Zen Peng, Marina V. Rodnina, Niels Fischer

**Affiliations:** 1 Project Group Molecular Machines in Motion, Department of Physical Biochemistry, Max Planck Institute for Multidisciplinary Sciences, Göttingen, Germany; 2 Department of Physical Biochemistry, Max Planck Institute for Multidisciplinary Sciences, Göttingen, Germany; 3 Present Address: Insempra GmbH, Planegg, Germany

**Keywords:** Cryoelectron microscopy, Ribosome

## Abstract

The selection of an open reading frame (ORF) for translation of eukaryotic mRNA relies on remodeling of the scanning 48S initiation complex into an elongation-ready 80S ribosome. Using cryo-electron microscopy, we visualize the key commitment steps orchestrating 48S remodeling in humans. The mRNA Kozak sequence facilitates mRNA scanning in the 48S open state and stabilizes the 48S closed state by organizing the contacts of eukaryotic initiation factors (eIFs) and ribosomal proteins and by reconfiguring mRNA structure. GTPase-triggered large-scale fluctuations of 48S-bound eIF2 facilitate eIF5B recruitment, transfer of initiator tRNA from eIF2 to eIF5B and the release of eIF5 and eIF2. The 48S-bound multisubunit eIF3 complex controls ribosomal subunit joining by coupling eIF exchange to gradual displacement of the eIF3c N-terminal domain from the intersubunit interface. These findings reveal the structural mechanism of ORF selection in human cells and explain how eIF3 could function in the context of the 80S ribosome.

## Abstract (web summary)

Cryo-electron microscopy reveals the mechanism of human translation initiation from codon scanning to subunit joining. The structures show the roles of the Kozak sequence, GTP hydrolysis by eukaryotic initiation factor 2 (eIF2) and eIF5B in 48S remodeling, as well as that of eIF3 in the control of 60S docking.

## Main

Translation in eukaryotes is largely regulated at the initiation stage. Translation initiation begins with the recruitment of eukaryotic initiation factors (eIFs), including eIF1, eIF1A and eIF3, and the ternary complex (TC) consisting of the translational guanosine triphosphatase (GTPase) eIF2, guanosine triphosphate (GTP) and methionyl initiator transfer RNA (Met-tRNA_i_^Met^) to the small ribosomal subunit (40S). This complex binds the 5′ end of the mRNA with the aid of eIF4F, forming the 48S initiation complex^1–5^. The 48S complex then scans along the mRNA in the 5′-to-3′ direction, searching for translation start codons, typically AUG, within the context of a Kozak sequence. The start codon recognition by Met-tRNA_i_^Met^ results in the eviction of eIF1 and recruitment of the GTPase-activating protein eIF5, which triggers GTP hydrolysis by eIF2, most likely through a classical arginine finger mechanism^6^. GTP hydrolysis by eIF2, in turn, triggers further remodeling of the 48S complex, ultimately facilitating the eIF5B-mediated docking of the large ribosomal subunit (60S).

The process of 48S remodeling is driven by several major commitment steps, including start codon recognition, GTP hydrolysis by eIF2, tRNA handover from eIF2 to eIF5B and eIF5B-mediated subunit joining. Pioneering structural studies in yeast and mammals provided first insights into start codon recognition, revealing that codon recognition induces a conformational change of the 48S from an open scanning state^7–10^ to a closed state^8,9^, leading to the release of eIF1 (refs. ^10,11^) and binding of eIF5 (refs. ^12,13^). Subsequent events of eIF5-induced GTP hydrolysis and phosphate (Pi) release from eIF2 commit the 48S to the selected start codon^14–16^ and facilitate eIF5B recruitment, triggering a cascade of rearrangements that prepare the 48S for subunit joining^14,17,18^. Although a number of 48S closed structures have been reported, most were captured after codon recognition but before GTP hydrolysis by eIF2. In contrast, the high-resolution details of codon reading by the open 48S, the events following GTP hydrolysis by eIF2 and eIF5B binding and the structural mechanism through which the multistep 48S remodeling process controls start codon selection and subunit joining remain poorly resolved.

Start codon selection depends on the nucleotide residues surrounding the AUG, referred to as the Kozak sequence. Specifically, the most critical residues are a purine at the −3 position (with the A of AUG being designated as +1) and a guanosine at the +4 position in mammals^19^. With AUG in a suboptimal context, the majority of 48S complexes do not initiate translation at the start site and continue scanning for downstream sites^19,20^. While the Kozak context is known to stabilize start codon recognition through local interactions within the closed 48S (ref. ^11^), its structural role in 48S remodeling and the molecular basis of sequence specificity remain unclear.

Another open question pertains to how the large multisubunit eIF3 complex regulates the docking of the 60S subunit, making the final commitment step of initiation toward elongation. eIF3 prevents premature 60S joining^7,10,21^ and earlier structural studies suggested that the entire eIF3 may dissociate to enable ribosomal subunit joining^18^. However, recent in vivo results challenge this assumption, indicating that eIF3 remains bound to the 40S until and possibly even after 60S joining and may function within the context of the elongating 80S ribosome^22–25^.

In this study, we visualize key intermediates along the human 48S remodeling pathway using single-particle cryo-electron microscopy (cryo-EM), offering a high-resolution perspective from the early start site sampling events to the formation of the complex ready for 60S subunit docking. Our structures elucidate the role of the Kozak sequence in 48S remodeling, reveal the elusive events that ensue after eIF5-induced GTP hydrolysis by eIF2 and subsequent binding of eIF5B and show how eIF3 controls ribosome subunit joining.

## Results

### Cryo-EM visualizes pathway of human 48S remodeling

**Fig. 1:**
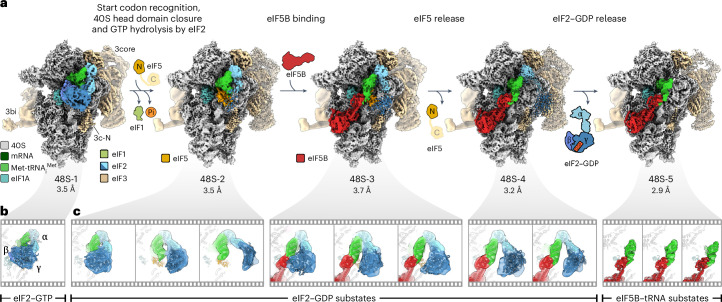
Pathway of human 48S remodeling from codon scanning to subunit joining. **a**, Cryo-EM maps of major states with distinct ligand composition. For each cryo-EM map, the overall resolution is given in Å and densities are rendered at 4*σ* (2.5*σ*–3.5*σ* for dynamic components). For eIF3b/i (3bi), the major conformational substates are shown for each state (Extended Data Figs. 1 and 3). Note the scattered densities for eIF2 in 48S-2 to 48S-4 even at low density thresholds, indicating the substantial dynamics of eIF2–GDP. 3core, octameric eIF3 core; 3c-N, NTD of eIF3c; N, NTD of eIF5; C, unresolved CTD of eIF5 (refs. ^43,46,47^). **b**, Close-up view of the eIF2–GTP–Met-tRNA_i_^Met^ TC in 48S-1. α, β and γ, subunits of eIF2; γ entails eIF2’s GTPase domain. **c**, Conformational substates of eIF2–GDP, Met-tRNA_i_^Met^ and eIF5B–GTP in 48S-2 to 48S-5 (Extended Data Fig. 2).

**Extended Data Fig. 1:**
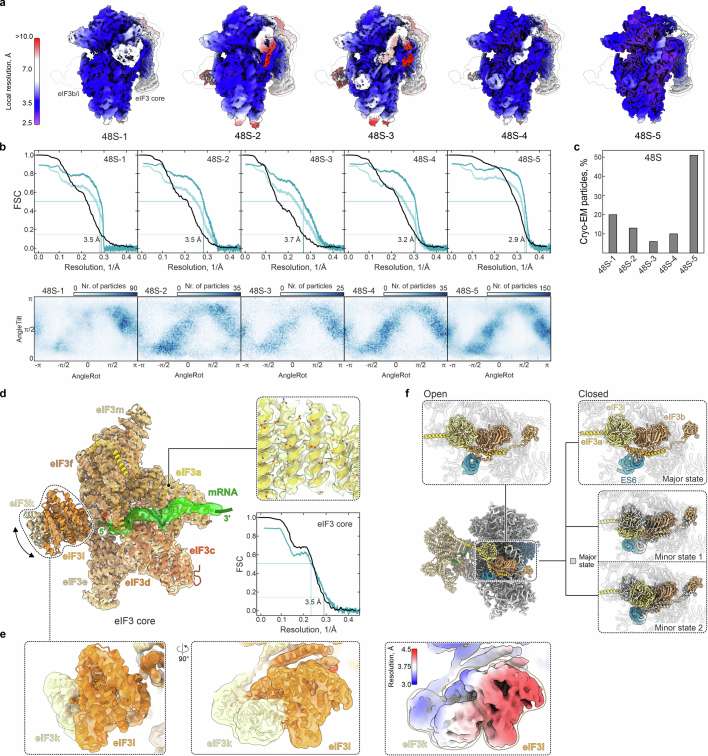
Cryo-EM reconstructions and eIF3 structure and dynamics. **a)** Local resolution estimation for the overall cryo-EM maps of 48S-1 to 48S-5. The lower local resolutions for individual subunits of eIFs reflect their conformational dynamics. Subsequent local sorting and/or refinement resolved the structures of dynamic eIFs (Extended Data Figs. 2, 3 and Methods). **b)** Fourier-Shell-Correlation (FSC) curves (top) and angular distributions of particles (bottom) for cryo-EM structures of 48S-1 to 48S-5. FSC plots show half-map FSCs (black), model-map FSCs (light blue) for the complete atomic models and model-map FSCs (blue) for the atomic models excluding the most dynamic eIFs resolved by subsequent image processing, that is eIF3 in 48S-1 and 48S-5, and eIF2 and eIF3 in 48S-2 to 48S-4. **c)** Cryo-EM particle populations for each 48S state. **d)** eIF3 core at 3.5 Å resolution and mRNA path in the closed 48S, obtained by focused refinement (Extended Data Fig. 3a and Methods). The eIF3k/l subunits are highly dynamic (as indicated by arrows). Particles of 48S-2 to 48S-5 were combined for the analyses of the eIF3 core and eIF3b/i subcomplex in the closed state. **e)** Structure of the eIF3k/l module in the closed state. Flexible refinement of the eIF3 core improved local resolution for dynamic eIF3k/l (Methods). **f)** Structure and dynamics of the eIF3b/i subcomplex and 18S rRNA expansion segment 6 (ES6) in the open and closed 48S obtained by focused classification (Extended Data Fig. 3a, c and Methods).

**Extended Data Fig. 2:**
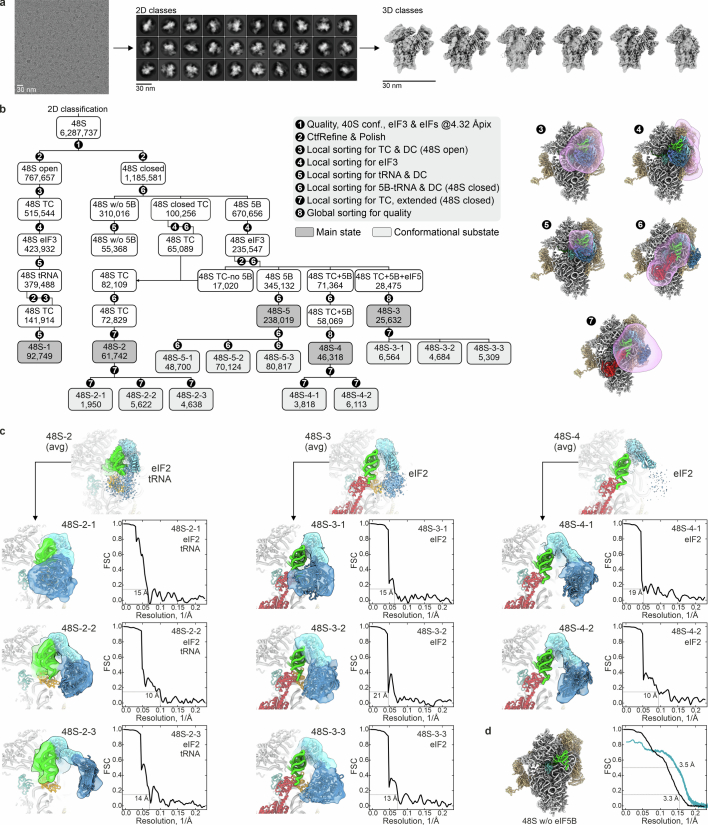
Cryo-EM analysis of human 48S complexes. **a)** Representative micrograph (left), 2D classes (center) and 3D classes (right) from the cryo-EM data. **b)** Computational sorting of cryo-EM data. Classification steps and masks (transparent pink) used for local sorting are indicated. Numbers denote the number of particles for the respective populations. Data processing was performed at the final pixel size of 1.16 Å/pixel, if not indicated otherwise. **c)** Focused classification strategy to resolve eIF2 and Met-tRNA_i_^Met^ dynamics. Average densities are shown for eIF2 in 48S-2 to 48S-4 (top). Below are local densities (left) and local FSC curves (right) for eIF2 substates obtained by focused classification. **d)** Structure of the late-stage 48S without eIF5B at 3.3 Å resolution. FSC curves (right) indicate half-map (black) and map-model (blue) FSCs.

To visualize the complete trajectory of 48S remodeling, we assembled human 48S complexes in vitro on mRNA with the AUG start codon and an upstream near-cognate AUC codon. The reconstitution assay included eIF1, eIF1A, the eIF2–GTP–Met-tRNA_i_^Met^ TC, eIF3, eIF5, eIF5B, eIF4A, eIF4B, eIF4E and eIF4G, along with adenosine triphosphate (ATP) and GTP. In contrast to previous structural work^7–13^, which used nonhydrolyzable GTP analogs and/or omitted eIF5 to avoid GTP hydrolysis, here, we added GTP and the late-stage remodeling factors eIF5 and eIF5B to visualize the events ensuing upon GTP hydrolysis by eIF2 and eIF5B binding on a fully assembled 48S. We acquired an extensive cryo-EM dataset of ~6,000,000 ribosome particle images to obtain high-resolution snapshots, including transient intermediates, such as the open 48S assembled on a near-cognate codon. Sorting of the cryo-EM data for compositional heterogeneity enabled us to visualize five intermediates on the initiation pathway at 2.9–3.7 Å resolution (48S-1 to 48S-5; Fig. 1a, Extended Data Figs. 1 and 2, Table 1 and Methods). All 48S intermediates contain eIF3, eIF1A, mRNA and Met-tRNA_i_^Met^ but differ in composition with respect to eIF1, eIF2, eIF5 and eIF5B. The stepwise change in eIF composition suggests the sequence of factor binding and dissociation events during 48S remodeling. It starts from the open scanning state (48S-1) through codon recognition and GTP hydrolysis by eIF2 (48S-2) to the subsequent binding of eIF5B (48S-3), followed by sequential release of eIF5 (48S-4) and eIF2 (48S-5), preparing the 48S for 60S joining (Fig. 1a).

**Table 1:** Cryo-EM structure determination

Ribosomal complex	48S-1	48S-2	48S-3	48S-4	48S-5	Off-pathway 48S without eIF5B	eIF3 core in closed 48S
EM Data Bank and PDB identifiers	EMD-17696, PDB 8PJ1	EMD-17697, PDB 8PJ2	EMD-17698, PDB 8PJ3	EMD-17699, PDB 8PJ4	EMD-17700, PDB 8PJ5	EMD-17701, PDB 8PJ6	EMD-19128, PDB 8RG0
**Data collection**							
Microscope	Titan Krios	Titan Krios	Titan Krios	Titan Krios	Titan Krios	Titan Krios	Titan Krios
Camera	Falcon III	Falcon III	Falcon III	Falcon III	Falcon III	Falcon III	Falcon III
Magnification	59,000	59,000	59,000	59,000	59,000	59,000	59,000
Voltage (kV)	300	300	300	300	300	300	300
Electron dose (e^−^ per Å^2^)	40–50	40–50	40–50	40–50	40–50	40–50	40–50
Defocus range (μm)	0.2–2.5	0.2–2.5	0.2–2.5	0.2–2.5	0.2–2.5	0.2–2.5	0.2–2.5
Pixel size (Å)	1.16	1.16	1.16	1.16	1.16	1.16	1.16
**Cryo-EM reconstruction**							
Initial particles (no.)	6,287,737	6,287,737	6,287,737	6,287,737	6,287,737	6,287,737	6,287,737
Final particles (no.)	92,749	61,742	25,632	46,318	238,019	55,368	371,711
Point group symmetry	*C1*	*C1*	*C1*	*C1*	*C1*	*C1*	*C1*
FSC threshold	0.143	0.143	0.143	0.143	0.143	0.143	0.143
Map resolution (Å)	3.5	3.5	3.7	3.2	2.9	3.3	3.5
Resolution metric	Gold-standard FSC	Gold-standard FSC	Gold-standard FSC	Gold-standard FSC	Gold-standard FSC	Gold-standard FSC	Gold-standard FSC
Map resolution range (Å)	2.9–18	3.0–21	3.0–19	2.9–18	2.6–17	2.8–23	3.0–10
Sharpening *B* factor (Å²)	−49.7	−44.5	−49.1	−26.2	−15.5	−35.4	−109.5
**Atomic model refinement**							
Resolution (Å)^a^	3.8 (3.5)	4.0 (3.5)	4.3 (3.7)	3.9 (3.3)	3.1 (3.0)	3.8	4.4
Cumulative RSCC (%) >0.8/>0.6/>0.4	0.64/0.8/0.89	0.60/0.75/0.84	0.58/0.75/0.84	0.60/0.73/0.82	0.60/0.75/0.84	0.57/0.72/0.81	0.80/0.96/0.99
Initial models used	Methods	Methods	Methods	Methods	Methods	Methods	Methods
MolProbity score	1.69	1.72	1.68	1.61	1.59	1.64	1.89
Clashscore	6.62	6.69	6.41	5.29	5.37	6.39	8.87
No. atoms/no. residues/RSCC							
Total	116,590/12,726/0.69	115,385/12,565/0.57	120,317/12,922/0.69	119,272/12,790/0.61	113,037/11,944/0.71	106,927/11,344/0.70	31,663/4,085/0.85
RNA	40,183/1,876/0.81	40,260/1,886/0.73	40,260/1,853/0.83	40,260/1,886/0.77	40,238/1,885/0.82	39,445/1,848/0.86	3,342/152/0.82
Proteins	76,266/10,850/0.67	75,018/10,679/0.57	79,917/11,069/0.68	78,864/10,904/0.60	72,546/10,059/0.68	67,393/9,407/0.66	28,229/3,933/0.86
Proteins without eIF3	47,059/5,946/0.84	46,331/5,851/0.78	51,225/6,470/0.87	50,172/6,337/0.83	44,648/5,592/0.89	39,482/4,940/0.90	5,148/650/0.89
eIF3 core (focused refinement)	27,177/3,911/0.71	-	-	-	-	-	22,933/3,283/0.87 (0.77)^b^
CC overall							
CC (mask)	0.68	0.67	0.67	0.67	0.75	0.62	0.75
CC (box)	0.73	0.83	0.82	0.81	0.81	0.81	0.86
*B* factors							
Protein	75.42	164.72	169.72	149.81	93.47	142.28	190.1
Nucleotide	116.15	166.92	196.06	147.86	111.68	160.62	158.3
Ligands and ions	57.75	105.72	118.85	102.87	68.32	92.70	138.9
Root-mean-square deviations							
Bond lengths (Å)	0.004	0.006	0.005	0.004	0.005	0.004	0.007
Bond angles (°)	0.769	1.177	0.948	0.902	0.731	0.778	1.352
Ramachandran plot							
Favored (%)	95.30	94.94	95.37	95.27	95.64	95.80	93.84
Allowed (%)	4.40	5.06	4.63	4.73	4.36	4.18	6.14
Disallowed (%)	0.00	0.00	0.00	0.00	0.00	0.01	0.03
Rotamer outliers (%)	0.00	0.00	0.05	0.00	0.00	0.00	0.08
Cβ outliers (%)	0.00	0.00	0.00	0.00	0.00	0.00	0.00
CaBLAM outliers (%)	3.36	3.17	3.15	3.17	3.16	2.99	3.66

FSC, Fourier shell correlation; RSCC, real-space correlation coefficient. For model refinement, final maps of 48S-1, 48S-2, 48S-4 and 48S-5 were resampled to 432 × 432 × 432 pixels (that is, a pixel size of 0.967 Å).

^a^Resolution at which map-model FSC = 0.5. In brackets, resolution when excluding most dynamic eIFs resolved by subsequent image analysis (that is, eIF3 for 48S-1 and 48S-5 and eIF2 and eIF3 for 48S-2 to 48S-4).

^b^RSCC of eIF3 core without eIF3k and eIF3l. In brackets, RSCC of eIF3k and eIF3l vs. 3DFlex map.

**Fig. 2:**
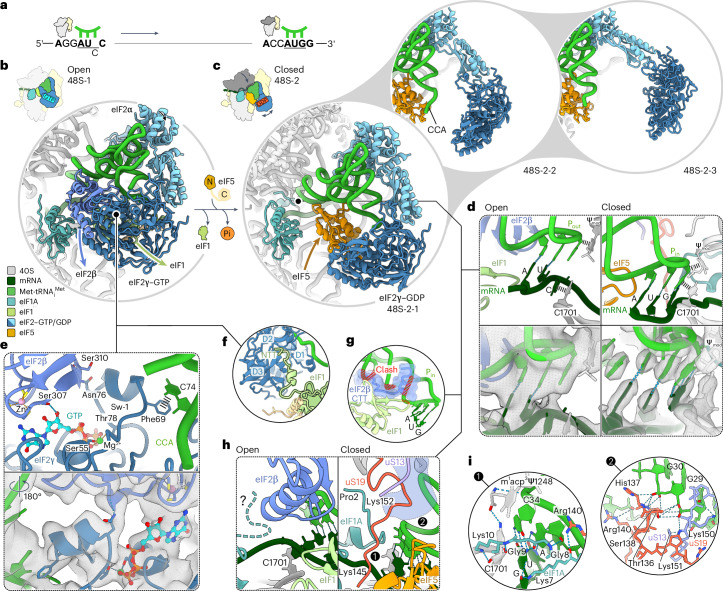
48S remodeling upon transition from codon scanning to start site commitment. **a**, Schematic of the 48S position on mRNA with Met-tRNA_i_^Met^ anticodon (green). The codon in the P site is underlined (that is, near-cognate AUC in open 48S-1 and AUG in closed 48S-2 to 48S-5). **b**,**c**, Close-up views of 48S-1 (**b**) and 48S-2 (**c**) showing changes (arrows) upon AUG recognition and eIF5-induced GTP hydrolysis and Pi release by eIF2. Note eIF2γ–GDP, which releases the tRNA acceptor stem partially in substate 48S-2-1 and completely in 48S-2-2 and 48S-2-3 (upper right panels). **d**, Remodeling of the decoding center. Left: partial codon–anticodon interaction of P_out_ tRNA with AUC. Right: AUG recognition by P_in_ tRNA. Ψ_mod_, m^1^acp^3^Ψ1248. Bottom: cryo-EM densities (gray mesh) for the respective codon–anticodon regions in open 48S-1 (left) and the best-resolved closed 48S, 48S-5 (right; density for Ψ_mod_ is shown at a lower threshold). **e**, GTPase domain of eIF2 with GTP and Met-tRNA_i_^Met^ in 48S-1 (top) and corresponding cryo-EM density (bottom). Sw-1, switch 1 of eIF2γ; CCA, tRNA CCA end. **f**, The NTT of eIF1 binds to domains 1 to 3 of eIF2γ (D1–D3), restricting conformational freedom of eIF2γ in the open 48S. **g**, P_in_ tRNA in closed 48S is incompatible with eIF1 binding and the eIF1–eIF2β-CTT interaction. **h**, Protein tails monitor AUG recognition. Left, unstructured eIF1A-NTT (‘?’) in the absence of full base pairing. Right, base pairing of the tRNA anticodon to AUG orders functionally important^16,30^ protein tails. The purple-shaded area denotes the former binding position of eIF2β, residue numbers indicate terminal residues and encircled numbers correspond to the close-up views in **i**. **i**, Interaction network of protein tails with the codon–anticodon helix in closed 48S (shown for 48S-5).

**Extended Data Fig. 3:**
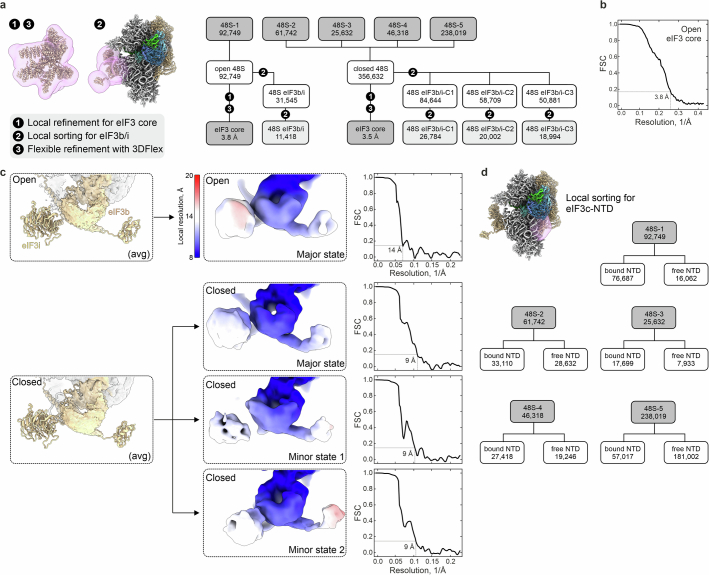
Cryo-EM analysis of eIF3 in open and closed 48S. **a)** Local refinements of the eIF3 core and local sorting for the eIF3b/i subcomplex in the open (48S-1) and closed states (48S-2 to 48S-5). The masks used for the respective steps are shown on the left. **b)** FSC curve for eIF3 core in open 48S obtained by focused flexible refinement. **c)** eIF3b/i in open and closed 48S, showing average densities for eIF3b/i (left), local resolution maps (center) and local FSCs (right) for eIF3b/i substates obtained after focused classification. **d)** Focused sorting for the NTD of eIF3c to determine the fraction of 40S-bound (“bound”) vs. unbound eIF3c-NTD (“free”).

Through additional sorting for conformational substates, we unraveled further dynamics within the complexes (Extended Data Figs. 1–3), revealing large-scale fluctuations of eIF2–GDP, the trajectory of Met-tRNA_i_^Met^ and the dynamics of eIF5B–GTP (Figs. 1b,c and 2 and Extended Data Fig. 2). Extensive sorting and focused refinements allowed us to fully resolve all eIF3 subunits (eIF3a–m^7^) except for eIF3j, which functions at an earlier step^14,26–28^. This approach also enabled visualizing the dynamics of the eIF3b/i subunits and tracing of the mRNA path along the eIF3 core up to mRNA residue −33 upstream of the AUG start codon (Extended Data Figs. 1d–f and 3a–c and Methods).

### Codon scanning by the open 48S

**Fig. 3:**
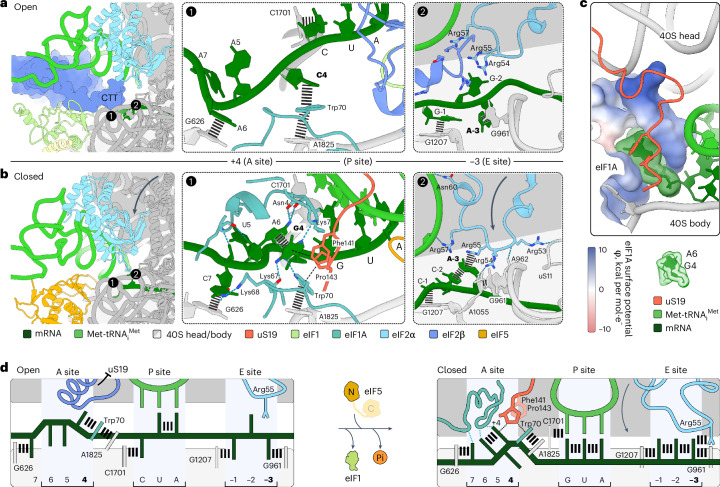
The role of start codon context in facilitating codon scanning and 40S closure. **a**,**b**, Interactions of mRNA nucleotides at +4 and −3 positions in the open 48S-1 (**a**) and in the closed posthydrolysis 48S-2 (**b**). Left: overview of the decoding center (encircled numbers refer to close-up views in the center and right panels). Center: the +4 base interactions in the A site. Note the compact mRNA configuration in closed 48S-2, stabilizing eIF1A-NTT and, thereby, 40S head closure, and the guanosine-specific interactions of G4. Right: interaction network of the −3 nucleotide in the E site. CTT, CTT of eIF2β blocking 40S head closure. **c**, mRNA–eIF1A-NTT network in the posthydrolysis 48S arresting the mRNA and stabilizing 40S head closure. For eIF1A, the surface potential is shown, illustrating its highly basic (blue) NTT. The NTT inserts between the 40S body and head domains and, together with uS19-CTT, encloses the mRNA. **d**, Schematic of Kozak sequence residues facilitating codon reading in the open 48S (left) and stabilizing 48S closure (arrow) in the posthydrolysis state (right). Interactions at the −3 position change upon AUG recognition and eIF1 release^11^, whereas Kozak +4 interactions are only changed in the posthydrolysis 48S. Key residues and positions appear in bold.

While moving along the mRNA 5′ untranslated region (UTR), 48S rapidly scans for potential start sites^2,19,29^. 48S-1 captures the open scanning 48S with eIF1, eIF1A and eIF2–GTP–Met-tRNA_i_^Met^ reading the AUC codon upstream of the canonical AUG (Figs. 2 and 3). In this conformation, the tRNA anticodon and the mRNA are shifted by ~10 Å out of the canonical P site on the 40S body domain into the P_out_ state (Fig. 2a,b,d). While the density indicates some mRNA flexibility, nucleotides U36 and A35 in the tRNA anticodon base pair with A and U of the mRNA, respectively. Although the next (nonmatching) nucleotide in the mRNA codon is within reach for base pairing, it flips away and stacks onto the universally conserved C1701 base of 18S ribosomal RNA (rRNA), whereas the ribose of the unpaired anticodon nucleotide C34 stacks onto the highly conserved hypermodified m^1^acp^3^Ψ1248 residue of 18S rRNA (Fig. 2d, left).

**Extended Data Fig. 4:**
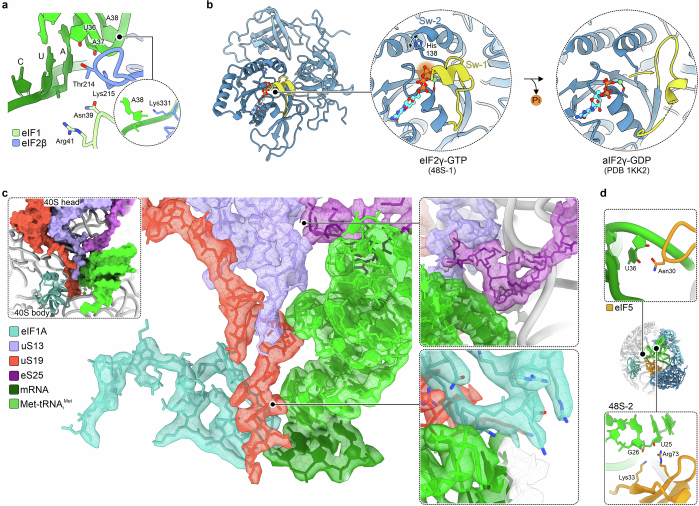
Details of codon scanning, AUG recognition and eIF2-GTP. **a)** Interactions of eIF1 and eIF2β with mRNA and tRNA backbone, respectively, in 48S-1. **b)** GTPase switch 1 (Sw-1) of eIF2γ with GTP (48S-1) and in the GDP-bound form (aIF2γ-GDP from *P. abyssi*^34^). The side chain of the conserved His138 in the switch 2 region (Sw-2) is unresolved in 48S-1, indicating flexibility. **c)** Protein tails enclosing the codon-anticodon duplex in the closed 48S-5. Left inset: Overview. Top right: Close-up of contacts between uS13-CTT, eS25-NTT and the ASL and variable loop region of Met-tRNA_i_^Met^. Bottom right: Close-up of interaction between eIF1A-NTT and the backbone of the tRNA anticodon. **d)** eIF5–tRNA interactions in 48S-2. Top: Asn30 reaches towards U36 of the tRNA anticodon. Center: Overview. Bottom: Lys33 and Arg73 reach the backbone of the tRNA’s D-arm.

eIF1 and eIF2β have crucial roles in stabilizing the labile codon-reading interaction by bridging between mRNA and Met-tRNA_i_^Met^ (Fig. 2d, left and Extended Data Fig. 4a), in line with the overall arrangement in earlier structures^8–10^. The potential stabilizing interactions involve eIF1 residue Arg41 reaching the backbone of the mRNA codon in the P site. Tyr214 and Lys215 of eIF2β extend toward the backbone of the tRNA anticodon stem loop (ASL) at U36 and A37 from the A site, while Lys331 of eIF2β reaches toward A38 from the E site. In the open state, the unstructured regions of ribosomal proteins, including the functionally important N-terminal tail (NTT) of eIF1A (ref. ^30^), appear to be highly flexible. Importantly, eIF2β obstructs the ribosomal protein tails from tRNA binding in the open state, thereby helping to prevent premature 40S head domain closure in the absence of cognate codon–anticodon interaction (Fig. 2h, left).

The α, β and γ subunits of eIF2 encase and stabilize Met-tRNA_i_^Met^ in the P_out_ state, facilitating processive mRNA scanning^8–10^ (Fig. 2b). In this conformation, eIF2α and eIF2β interact with the tRNA body from opposite sides, with domain 1 of eIF2α anchoring eIF2 to the 40S head. By sorting for the flexible eIF2γ subunit, we obtained the structure of eIF2γ bound to GTP, revealing that GTP binding stabilizes the compact state of the switch 1 region of eIF2γ, crucial for Met-tRNA_i_^Met^ binding (Fig. 2e, Extended Data Fig. 4b and Methods). Met-tRNA_i_^Met^ binding is stabilized by stacking interactions between Phe69 and C74 in the tRNA CCA end; additionally, eIF2β contributes to stabilizing the compact switch 1 by interactions of its Ser310 with Asn76 of eIF2γ (Fig. 2e). The helix–turn–helix motif of eIF2β stabilizes the open state by interacting with both eIF1 and Met-tRNA_i_^Met^. The C-terminal tail (CTT) of eIF2β extends across eIF1 beneath the tRNA ASL toward the E site, where it interacts with eIF2α (Figs. 2g and 3a, left). This interaction expands the contact interface of eIF2β with eIF1 and creates an additional wedge that prevents premature tRNA and 40S head movement into the closed state (Fig. 2g). These findings align with the important roles of eIF1 and eIF2β in maintaining the fidelity of start site selection^8–10,30–32^. Moreover, we find that, in open 48S, the NTT of eIF1 reaches from the decoding center over the α-helix in eIF3c’s linker domain to the interdomain interface of eIF2γ, where it bridges all three domains of eIF2γ (Fig. 2f), thereby restricting the conformational freedom of eIF2γ and contributing to stabilization of the eIF2–GTP–Met-tRNA_i_^Met^ complex for scanning. The resemblance in the overall arrangements of eIF2β-CTT and eIF1-NTT in the human 43S open state before mRNA recruitment^28^, along with their sequence conservation in both human and yeast, indicates that both protein tails likely serve a similar functional role in stabilizing the open state at various stages of initiation.

**Extended Data Fig. 5:**
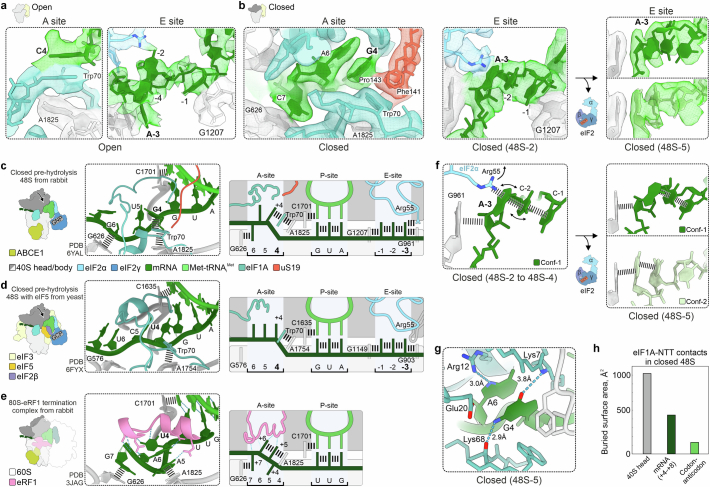
mRNA context interactions and dynamics. **a)** Experimental densities for interactions around mRNA nucleotides +4 and -3 in the open 48S-1. **b)** Experimental densities for mRNA interactions in closed 48S at the +4 position (left, shown for 48S-5), and at the -3 position in presence of eIF2 (center, shown for 48S-2) and after eIF2 release in 48S-5 (right); the latter density can be best explained by two alternative mRNA conformations. Density for G1207 was omitted in right panels. **c)** and **d)** Interactions of Kozak sequence residues in reported **c)** closed pre-hydrolysis 48S structures, that is mammalian 48S after eIF1 dissociation^11^, and **d)** the yeast 48S with eIF5^12^. Left: Schematic of the complexes. Center: Close-up of interactions in the A site. Right: Schematic of interactions in A, P and E sites. 18S rRNA and protein residues for complexes from rabbit in c) and e) reflect human numbering for better comparison. **e)** Compact mRNA conformation in the A site induced upon stop codon recognition by eukaryotic release factors in the 80S ribosome^39^. eRF1, eukaryotic release factor 1; mRNA positions +4 to +6, UAA stop codon. **f)** mRNA dynamics at the mRNA –3 position in closed 48S. Note the destabilization of mRNA conformation 1 (“Conf-1”) upon eIF2 release, which results in two alternative mRNA conformations in 48S-5 (“Conf-1” and “Conf-2”). **g)** Specific interactions of the key Kozak residue +4 guanosine, shown for 48S-5. The sidechain of Glu20 is within reach of the guanosine’s amino group, but only partially resolved, indicating a transient interaction. **h)** Buried surface area of eIF1A-NTT indicating the extent of its interactions in the closed post-hydrolysis 48S with 40S head, A-site mRNA, and the codon-anticodon duplex, respectively. Buried surface area was computed using ChimeraX 1.7^59^, averaging the values obtained for 48S-2 to 48S-5.

Unexpectedly, our structures reveal that the mRNA residues within the Kozak sequence^19^ contribute to start site selection during mRNA scanning by enhancing the stability of codon–anticodon interactions in the open state (Fig. 3a,d and Extended Data Fig. 5a). Specifically, the mRNA pyrimidine adjacent to the AUC codon at the +4 position forms stacking interactions with Trp70 of eIF1A, which is further stabilized by stacking onto the universally conserved A1825 of 18S rRNA. A similar interaction pattern is observed in the closed mammalian 48S trapped after eIF1 release but before eIF5 binding and GTP hydrolysis (Extended Data Fig. 5c)^11^. Notably, in the open 48S, the functionally important purine at the mRNA −3 position^19^ stacks with its base onto G961 of 18S rRNA, while the mRNA base at the −2 position is within hydrogen-bonding distance of eIF2α Arg55 and the −1 base stacks onto G1207 of 18S rRNA (Fig. 3a, center and 3d, left). As a result, the mRNA codon in the P site is stabilized in the open state through stacking interactions at the critical −3 and +4 positions, which is likely to form more stable interactions with purines^19^. In summary, the stable interaction network involving eIF1, eIF2–GTP and Met-tRNA_i_^Met^ locks the 40S head in the open conformation and the tRNA in P_out_ for processive scanning along the mRNA. mRNA scanning, in turn, appears to be modulated by mRNA context, with favorable Kozak elements slowing down scanning by the open 48S to facilitate start codon selection.

### Start site commitment upon GTP hydrolysis by eIF2

The majority of 48S complexes progress beyond the near-cognate AUC codon to the downstream AUG, resulting in start codon recognition and GTP hydrolysis by eIF2 in 48S-2. AUG recognition induces the 40S head domain closure, shifting Met-tRNA_i_^Met^ and mRNA toward the 40S body^8–11^ in 48S-2 to 48S-5 (Fig. 1). Comparing our high-resolution structure of the open 48S-1 with the closed 48S structures, both before^8–13^ and after GTP hydrolysis (48S-2; Fig. 2a–d,h), highlights the intricate remodeling process resulting from irreversible GTP hydrolysis, which ultimately completes the selection of the start site.

In the open 48S-1, eIF1–eIF2β and eIF1 block 40S head closure and P_in_ formation (Fig. 2g). Upon transition to 48S-2, eIF1 is ejected^10,11^ and the N-terminal domain (NTD) of eIF5 takes the place of eIF1 (refs. ^12,13^), establishing multiple interactions with the P_in_ tRNA through highly conserved residues (Asn30, Lys33 and Arg73), which reach toward the tRNA anticodon and ASL region (Extended Data Fig. 4d). C1701 of the 40S body changes stacking interactions from the mRNA +3 base to tRNA base C34 (Fig. 2d). The hypermodified m^1^acp^3^Ψ1248 base of the 40S head guides Met-tRNA_i_^Met^ from P_out_ to P_in_ by maintaining stacking interactions with the ribose of tRNA residue C34, as in the prehydrolysis 48S (refs. ^11,12^). In 48S-5, the m^1^acp^3^Ψ1248 modification additionally stabilizes the stacking interactions and the cognate duplex by forming hydrogen bonds with tRNA backbone of C34 and C1701 base of 40S (Fig. 2i, left). Loss of this highly conserved hypermodification is linked to colorectal cancer and translation reprogramming^33^.

**Extended Data Fig. 6:**
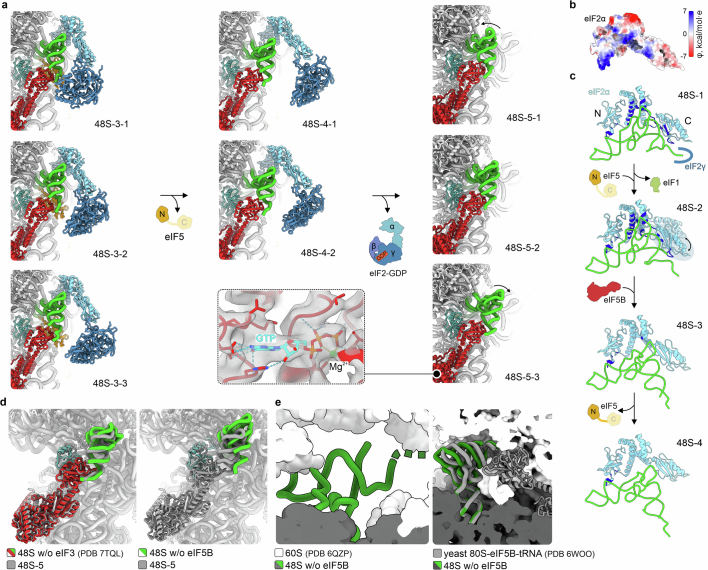
Dynamics of eIF–tRNA interactions and Met-tRNA_i_^Met^ positioning upon subunit joining. **a)** Structural dynamics of eIF2–GDP, eIF5B and Met-tRNA_i_^Met^ in substates of 48S-3 to 48S-5. Inset at the bottom, center: Cryo-EM density of the nucleotide binding pocket of eIF5B with native GTP in 48S-5. **b)** Local electrostatic potential of eIF2α, indicating basic patches (blue) that support tRNA binding, cf. panel c). **c)** Changes in eIF2α-Met-tRNA_i_^Met^ interactions upon eIFs exchange. eIF2α contacts with the tRNA are highlighted in dark blue. N and C, NTD and CTD of eIF2α, respectively. **d)** Arrangement of Met-tRNA_i_^Met^ in 48S-5 (grey) vs. the previous 48S without eIF3^18^ (left) and vs. the present off-pathway 48S with tRNA in the absence of eIF5B (right). **e)** The orientation of Met-tRNA_i_^Met^ in the 48S without eIF5B is compatible with 60S joining (left) and differs only slightly from the yeast 80S subunit joining complex^65^ (right). The 48S was docked onto the 60S of the human 80S ribosome^49^, based on the subunit-arrangement in the yeast 80S subunit joining complex^65^.

Recruitment of eIF5 activates rapid GTP hydrolysis and Pi release by eIF2 (refs. ^14–16^), triggering a dramatic remodeling of the complex in 48S-2 (Fig. 2). In previous 48S structures with eIF5, eIF2 is trapped in the prehydrolysis state by a nonhydrolyzable GTP analog and most of the interactions of eIF2 with the tRNA are maintained upon eIF5 binding^12,13^. GTP hydrolysis triggers refolding of the GTPase switch 1 region (Extended Data Fig. 4b)^34^ and eIF2–GDP releases the tRNA CCA end (Fig. 2c), consistent with the substantially reduced affinity of eIF2–GDP to Met-tRNA_i_^Met^ (ref. ^35^). As a result, eIF2–GDP loses a crucial anchor point and becomes highly dynamic. eIF2α remains the only connection to the 40S, while eIF2β dissociates from the decoding center and eIF2γ samples a broad range of conformations away from the 40S (Figs. 1c and 2c and Extended Data Fig. 6a), which explains the destabilization of eIF2 binding to 40S after GTP hydrolysis^14,16^. The observed release of Met-tRNA_i_^Met^ from eIF2 and the resulting eIF2–GDP dynamics preclude the reversal of start codon recognition^16^, providing the structural basis for start site commitment upon GTP hydrolysis.

Upon AUG recognition and GTP hydrolysis, protein tails in the decoding center become structured, contributing to the stabilization of the closed state in 48-2 to 48S-5 (Fig. 2d,h,i and Extended Data Fig. 4c). The NTT of eIF1A is essential for ensuring accurate start codon selection^36^. In our 48S-2 to 48S-5 structures, we fully resolved the NTT of human eIF1A up to Pro2. The globular domain of eIF1A binds to the A site, while the NTT of human eIF1A forms a network of interactions that stabilize the cognate mRNA–tRNA duplex in the P site through RNA backbone interactions with Lys7, Gly8 and Gly9 (Fig. 2i, left). The arrangement of eIF1A’s globular domain and the interactions of its NTT with the codon–anticodon duplex are similar to those in the mammalian 48S without eIF1 and eIF5 (ref. ^11^) and in the yeast prehydrolysis 48S with eIF5 (ref. ^12^), while the human prehydrolysis 48S with eIF5 has a distinct eIF1A-NTT arrangement^13^. In contrast, in our previous closed human 48S structure, which was captured before eIF5 binding, eIF1A adopts a different orientation in the A site and eIF1A-NTT does not fully reach the codon–anticodon duplex in the P site, consistent with increased eIF1A dissociation rates at that stage^10^.

Dissociation of eIF2β from the decoding center in 48S-2 to 48S-5 makes place for the CTTs of ribosomal proteins uS19 and uS13 (Fig. 2h), which are fully resolved in our posthydrolysis structures (Extended Data Fig. 4c). The CTT of uS19 bridges the 40S head to the ASL of the tRNA, where it intertwines with uS13-CTT, forming a connection over the anticodon region and extending down to the mRNA in the A site (Fig. 2h, right). uS13-CTT additionally interacts with the ASL region and with the NTT of eS25, which in turn contacts the variable loop region of Met-tRNA_i_^Met^ (Extended Data Fig. 4c). A similar overall arrangement, but with distinct interactions of uS13-CTT and uS19-CTT, is observed in the mammalian prehydrolysis 48S complex when eIF1 and eIF5 are absent^11^. In contrast, in the yeast and human prehydrolysis 48S with eIF5, the movement of eIF2β is too small to allow uS19-CTT to access the decoding center and Met-tRNA_i_^Met^, whereas uS13-CTT and eS25-NTT seem to be flexible and do not reach the tRNA^12,13^. These results suggest that eIF2β has a role in controlling the access of uS19 to the decoding center upon 48S remodeling. Thus, eIF2β, in coordination with ribosomal protein tails, modulates the stability of start codon recognition, ensuring locking of the ribosome on the start codon after GTP hydrolysis has taken place.

### Kozak +4 residue in shaping the A-site mRNA and 40S closure

In the posthydrolysis structures 48S-2 to 48S-5, we observe large-scale remodeling of the mRNA conformation and its interactions in the A site (Fig. 3 and Extended Data Fig. 5). The recognition pattern of the crucial +4 purine in the Kozak sequence changes from a triple stack found in the open 48S-1 (Fig. 3a) and the closed prehydrolysis 48S (ref. ^11^) to an arrangement of three double stacks involving mRNA bases (G4–A6), 18S rRNA and eIF1A (A1825–Trp70) and uS19 (Pro143–Phe141), connected through CH–*π* interactions by Pro143 of uS19 (Fig. 3b). uS19 stabilizes the 40S domain closure by bridging between the 40S head and body (Fig. 3b,c), in line with its important role in +4 nucleotide recognition^16^.

The other key functional residue of the Kozak sequence, the purine nucleotide at the −3 position in the E-site mRNA, is engaged in stacking contacts with C-2 of mRNA, 18S RNA (G961) and eIF2α (Arg55) in 48S-2 to 48S-4 (Fig. 3b, right). Its position in 48S-2 does not change compared to the closed prehydrolysis 48S (ref. ^11^) (Extended Data Fig. 5c) but differs from the open 48S-1 (Fig. 3a, right). Position −3 is part of a network supporting eIF2α, which contacts the 40S head, P_in_ tRNA and the 40S body (A1055 of 18S rRNA and uS11) and stabilizes 40S head closure (Fig. 3b, right). Upon further remodeling, the dissociation of eIF2 in 48S-5 disrupts the interaction between eIF2α and the −3 mRNA base, resulting in two alternative mRNA conformations (Extended Data Fig. 5b,f). The maintenance of stacking interactions at key −3 and +4 positions throughout 48S remodeling explains the strong preference for purines at both positions in the Kozak sequence (Fig. 3d). In contrast, pyrimidines at the −3 and +4 positions destabilize these stacking interactions, as seen in the structure of the human prehydrolysis 48S with eIF5 (ref. ^13^), in agreement with the lower translation efficiency for the suboptimal Kozak sequence^19^.

Alterations in Kozak +4 base interactions result in compaction of the mRNA, accommodating four nucleotides in the A site instead of three (Fig. 3a,b,d). In open 48S-1 and the closed prehydrolysis 48S (ref. ^11^), the mRNA assumes a slightly bent conformation, with the +4 nucleotide stacking onto Trp70 of eIF1A, while the +6 base stacks onto the universally conserved base G626 of 18S rRNA (Fig. 3a, center and Extended Data Fig. 5b,c). In the posthydrolysis structures, the +6 position moves away and stacks on the reoriented +4 purine, pulling the +7 position as the fourth nucleotide into the A site, which in turn stacks on the vacated G626 (Fig. 3b, center). The resulting sharply bent mRNA configuration forms a tight interaction network with eIF1A in all posthydrolysis states (48S-2 to 48S-5), which is not found in prehydrolysis 48S structures. In 48S-2 to 48S-5, eIF1A-NTT and the globular OB domain of eIF1A encircle positions +4 and +6, engaging with the nucleotide bases (Lys7 and Lys67 or Lys68 with the G4 base; Asn4, Lys7 and Arg12 with the A6 base) and backbone (Lys7 with the G4 and C5 backbone). The extensive eIF1A–mRNA network stabilizes the closed 48S conformation, while the positively charged eIF1A-NTT fills the void between 40S head and body (Fig. 3c). These findings suggest that eIF1A-NTT may work by recognizing (and stabilizing) 40S closure and mRNA context, rather than by directly monitoring the codon–anticodon duplex (Extended Data Fig. 5h). Amino acid substitutions in this region of eIF1A-NTT have been implicated in cancer^37^.

In yeast, eIF1A-NTT also contributes to the stabilization of the closed 48S but the mRNA conformation and interactions within the A site differ from those observed in the mammalian structure (Extended Data Fig. 5d)^12^, reflecting the prehydrolysis state and the preference for U at the Kozak +4 position in yeast^38^. Human eIF1A recognizes specific features of the +4 guanosine base, in line with the preference for guanosine in the vertebrate Kozak sequence. The amino group at C2 of the guanosine base interacts with the backbone oxygen of either eIF1A Lys67 or Lys68 and is within reach of the Glu20 side chain, while the carbonyl group’s oxygen at C6 additionally interacts with the backbone amine of Lys7 in 48S-4 and 48S-5 (Fig. 3b, center and Extended Data Fig. 5g).

The conformation of eIF1A-stabilized compact mRNA in 48S-2 to 48S-5 resembles that in mammalian 80S termination complexes, where eukaryotic release factors induce a similarly compact yet structurally distinct A-site mRNA conformation upon stop codon recognition^39,40^ (Extended Data Fig. 5e). However, the mRNA–eIF1A network appears to be less rigid, with a strong preference at just one position in the Kozak sequence. The compact mRNA stabilized by eIF1A-NTT may function as a roadblock contributing to start site commitment in the posthydrolysis 48S.

### Met-tRNA_i_^Met^ handover and eIF release upon eIF5B binding

**Fig. 4:**
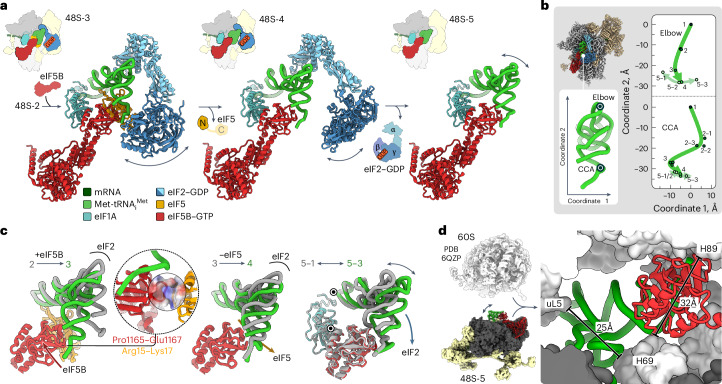
Met-tRNA_i_^Met^ transfer to eIF5B, eIF release and tRNA trajectory during translation initiation. **a**, Choreography of eIF5B binding and release of eIF5 and eIF2. The dynamics of eIF2–GDP enables eIF5B to bind before eIF2 is released and directs the tRNA transfer from eIF2 to eIF5B in 48S-3. The dissociation of eIF5 and eIF2 is sequential, in contrast to yeast, where they may leave together as a complex^48^. Arrows point to the flexibility of eIF2–GDP and Met-tRNA_i_^Met^, as identified through the resolution of multiple substates (Extended Data Fig. 6a). **b**, Met-tRNA_i_^Met^ trajectory measured relative to 48S-1 (‘1’) at the tRNA elbow (P atom of C56) and lower part of the acceptor stem (‘CCA’; P atom of A73). **c**, eIF5B binding and eIF5 and eIF2 release promote stepwise reorientation of Met-tRNA_i_^Met^. Inset: interaction of eIF5B with eIF5 in 48S-3. eIF5-NTT forms a positively charged cradle to support the negatively charged loop of eIF5B-CTD. Numbers denote 48S states; black dots denote the pivots for tRNA dynamics after eIF2 release in 48S-5 substates. **d**, The flexibility of Met-tRNA_i_^Met^ in 48S-5 (double-headed arrow) facilitates overcoming constriction sites during ribosomal subunit joining (Supplementary Video 1). Close-up view shows the eIF5B–Met-tRNA_i_^Met^ orientation in 48S-5 fitting into the 60S (ref. ^49^) within the 80S ribosome; bars indicate constriction sites. H69, H89, helices of 28S rRNA.

eIF5-induced GTP hydrolysis by eIF2 results in release of the tRNA CCA end from eIF2, paving the way for eIF5B recruitment (48S-2; Fig. 2c). Subsequent remodeling of the 48S complex upon eIF5B binding orchestrates the handover of Met-tRNA_i_^Met^ from eIF2 to eIF5B and the release of eIF5 and eIF2 (48S-3 to 48S-5; Fig. 4a,b and Extended Data Fig. 6). These events facilitate 60S subunit joining, making crucial steps toward the formation of the functional 80S elongation complex.

The 48S-3 structure reveals that eIF5B joins the complex after GTP hydrolysis by eIF2 but before the release of eIF5 and eIF2, which ensures a direct handover of the Met-tRNA_i_^Met^ CCA end from eIF2 to eIF5B. This observation is consistent with the single-molecule fluorescence resonance energy transfer data^18^ and crosslinking experiments^16^, countering models proposing that eIF5B binds to the 40S subunit only after eIF2 release^18^ or at an earlier stage of initiation before mRNA recruitment to the 43S preinitiation complex^41^. In 48S-3, the eIF5-NTT interacts with the C-terminal domain (CTD) of eIF5B, positioning the eIF5B-CTD for handover of the tRNA (Fig. 4b,c). eIF5B binding disrupts eIF2α-CTD interactions with the tRNA in all 48S-3 substates (Fig. 4a and Extended Data Fig. 6a,c), explaining why eIF5B promotes dissociation of eIF2 from the 48S (ref. ^16^). In 48S-4, eIF5 release allows the eIF5B–tRNA complex to relax toward its final position, now defined by the interactions of eIF5B-CTD with eIF1A and of the tRNA body with eIF2α-NTD (Fig. 4a–c and Extended Data Fig. 6a,c). The direct transfer of Met-tRNA_i_^Met^ from eIF2 to eIF5B in 48S-3 shields the tRNA CCA-Met moiety from the solvent (Fig. 4c, left), preventing spontaneous hydrolysis of Met-tRNA_i_^Met^ (ref. ^42^).

The release of eIF2 in 48S-5 has a minimal effect on the CCA end of the tRNA anchored to the eIF5B-CTD (Fig. 4a,b) but greatly increases tRNA flexibility by eliminating the contact between eIF2α-NTD and the tRNA body (Extended Data Fig. 6c). The unconstrained tRNA elbow samples a broad range of orientations (Fig. 4b,c and Extended Data Fig. 6a). Previous studies suggested that eIF1A–eIF5B–tRNA interactions have a crucial role in facilitating rapid 60S subunit joining by reorienting the tRNA to navigate through constriction sites on the way to 80S formation^18^. To test the structural importance of these interactions, we also determined the structure of an off-pathway 48S intermediate with eIF1A, eIF3 and Met-tRNA_i_^Met^ without eIF5B (Methods). Although the orientation of Met-tRNA_i_^Met^ in the complex without eIF5B differs from that in 48S-5, the inherent flexibility of free Met-tRNA_i_^Met^ would still allow for 60S joining (Extended Data Fig. 6d,e, Supplementary Video 1 and Methods). This is consistent with kinetic data suggesting that eIF5B mutants interfering with interactions between eIF5B and the tRNA’s CCA end have a relatively mild effect on the rate of eIF5B-mediated subunit joining^18^. Thus, while the contributions of eIF5B to subunit joining through positioning the Met-tRNA_i_^Met^ CCA end^18^ and shielding it from hydrolysis^42^ may be moderate, they nonetheless synergize with eIF5B’s other functions. These include providing a docking surface crucial for forming ribosomal intersubunit bridges^17^ and modulating the flexibility of the tRNA elbow region, which may additionally help the 60S subunit to accommodate the tRNA upon docking (Fig. 4d and Supplementary Video 1), together ensuring rapid transition toward 80S ribosome formation.

### How eIF3 controls ribosomal subunit joining

**Fig. 5:**
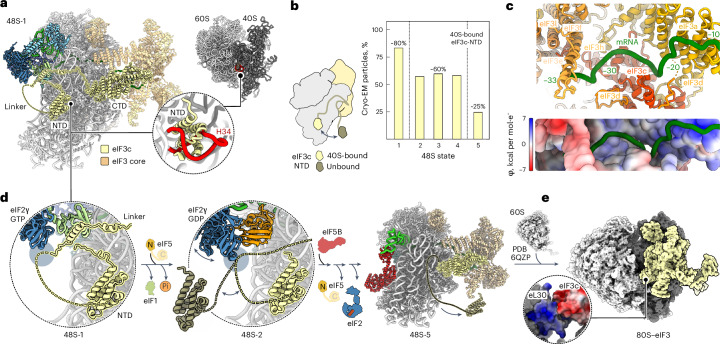
eIF3 as a sensor of the 48S state controlling 60S subunit joining by eIF3c. **a**, eIF3c-NTD sterically blocks subunit joining. Left: eIF3c interaction network in 48S-1. Right: close-up view showing the clash of eIF3c-NTD with 28S rRNA helix 34 (H34) of the 60S in context of the 80S ribosome^7,10^ (PDB 6QZP)^49^. The NTD, linker and CTD of eIF3c are indicated. **b**, eIF3c-NTD binding to the 40S depends on the 48S state. Left: model of eIF3c-NTD in 40S-bound and unbound state. Right: 40S-bound eIF3c-NTD population as a function of 48S state. Dashed lines indicate the substantial changes upon (1) codon recognition and eIF2 GTP hydrolysis and (2) eIF2 dissociation. **c**, mRNA path on eIF3. Top: mRNA 5′ end interactions with eIF3 subunits. Bottom: charge complementarity of eIF3 with mRNA path stabilizing eIF3 on the 40S (ref. ^21^). **d**, Structural coupling of 48S state and eIF3c, as visualized in the decoding centers of 48S-1 (left) and 48S-2 (center), and in the overall structure of 48S-5 (right). **e**, Model of the 80S ribosome–eIF3 complex after subunit joining based on docking 48S-5 onto human 80S ribosome structure^49^. Close-up view shows the charge complementarity of 60S protein eL30 (blue, basic) and eIF3c-CTD (red, acidic).

The multisubunit eIF3 complex serves as a platform for the eIF4F complex^7,13^ and prevents 60S joining^21^. Our structures demonstrate the persistent binding of eIF3 to the final 48S-5 complex (Fig. 1). This observation supports the idea that eIF3 remains associated during the transition from initiation to elongation, in line with the notion of eIF3 remaining on the 80S well into the elongation phase of translation^22–25^. In all mammalian 48S intermediates, the eIF3 core maintains a similar structure and location on the solvent side of the 40S platform (Fig. 1)^7,10,11,13^, stabilized by the mRNA interactions^21^ through extensive charge complementarity (Fig. 5c). Despite the dynamic nature of the eIF3b/i subcomplex, it does not extend to the intersubunit site of the 40S (Extended Data Fig. 1f), indicating that, unlike in the yeast system^9^, the mammalian eIF3b/i subcomplex does not have a direct role in controlling 60S joining. Instead, the NTD of eIF3c in the human 48S obstructs premature 60S association by blocking the 40S interface (Fig. 5a)^7,10^.

**Extended Data Fig. 7:**
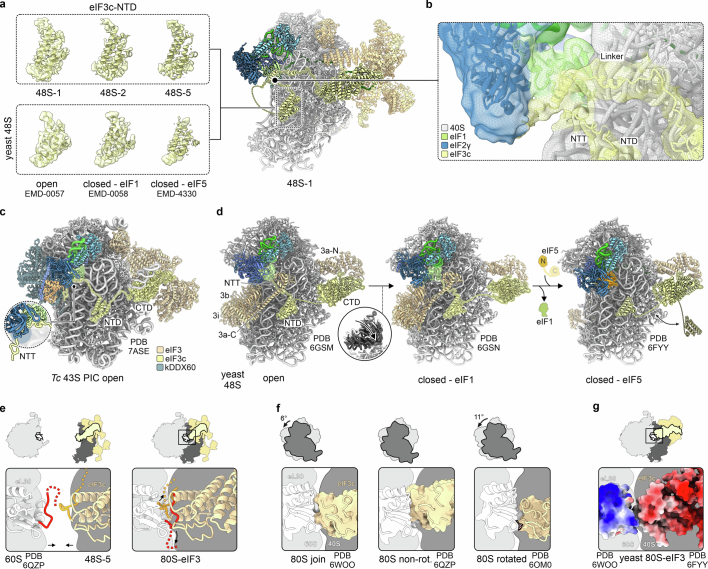
Conserved mechanism of eIF3c-NTD mediated coupling between 48S remodeling and 60S subunit joining. **a)** Cryo-EM density of eIF3c-NTD in the present structures (top) and in reported yeast 48S complexes^9,12^ (bottom, with EMDB codes). **b)** eIF3c linker and NTT in 48S-1. The low-pass filtered density map (to 6 Å) indicates an interaction between the eIF3c-linker region and eIF2γ and enabled modeling the NTT-backbone of eIF3c (starting from residue ~34). **c)** Reported structure of the *Trypanosoma cruzi* (*Tc*) 43S pre-initation complex^43^. The kinetoplastid-specific kDDX60 helicase stabilizes the eIF-interaction network, revealing an overall eIF3c-interaction network similar to yeast 48S. The interaction between eIF2γ and the eIF3c-NTT is clearly seen (inset, blue disc). In kinetoplastids and yeast, eIF3c-NTT appears to take over the function of the eIF3c-linker in the human system. **d)** Comparison to yeast 48S structures^9,12^, suggesting a similar mechanism of eIF3c controlled-subunit joining. Note the yeast eIF3a-CTD–eIF3b–eIF3i sub-complex (eIF3a/b/i), which additionally blocks 60S joining (left and center). The anti-association activity of eIF3a/b/i may be also connected to the eIF3c-NTT, which appears to interact with eIF3b in the yeast 48S in an eIF1-dependent manner. Replacement of eIF1 by eIF5 abolishes interaction of eIF3c-NTT with eIF1 and with eIF3b, resulting in retraction of eIF3a/b/i from the intersubunit interface (right). 3a-N, NTD of eIF3a; 3a-C, CTD of eIF3a; 3b, eIF3b; 3i, eIF3i. **e)** Compatibility of eL30 and eIF3c with 60S^49^ docking. The human eL30-CTT (red) and the eIF3c linker region (yellow) are inherently flexible, compatible with eIF3c binding. **f)** Modeling 60S-eIF3 interface dynamics during translation. 48S-5 was docked onto the 60S based on the subunit arrangements upon subunit joining^65^ (“80S join”, left), in the non-rotated ground state^49^ (“80S non-rot.”, center) and the rotated translocation state^67^ (“80S rotated”, right). The rotated state results in a minor clash of eL30 and eIF3c (red area), which might be absorbed by protein flexibility. **g)** Model of the yeast 60S-eIF3 interface. The closed yeast 48S with eIF3 and eIF5 bound^12^ fits with the yeast 60S^65^ and suggests a charge-complementary contact between eL30 (blue, basic) and eIF3c (red, acidic).

Upon comparing 48S states, we noted a progressive reduction in eIF3c-NTD occupancy at the 40S interface throughout 48S remodeling, as manifested by a deterioration in the quality of the eIF3c-NTD density in the respective cryo-EM maps (Extended Data Fig. 7a). This was quantified through local classification of the cryo-EM data (Fig. 5b and Extended Data Fig. 3d). In the open 48S-1, eIF1 stabilizes eIF3c-NTD on the 40S by interacting with an α-helical element in the eIF3c linker region (Fig. 5d), a feature also observed in the scanning 48S (ref. ^7^) and previous open 48S (ref. ^10^). Moreover, the eIF3c linker region also interacts with eIF2γ in 48S-1, which provides another anchor point contributing to eIF3c-NTD stabilization on the 40S at this stage (Fig. 5d, left and Extended Data Fig. 7b). Notably, *Trypanosoma cruzi* initiation complexes show a similar interaction of eIF2γ with eIF3c (ref. ^43^) (Extended Data Fig. 7c). In 48S-2, the anchor point to eIF1 is lost, as eIF1 is replaced by eIF5, which does not interact with the eIF3c linker, while eIF2γ becomes dynamic upon GTP hydrolysis, causing it to fluctuate away from the 40S. Together, these events enhance the diffusional freedom of eIF3c-NTD, explaining the substantial reduction in eIF3c-NTD occupancy on the 40S in 48S-2 to 48S-4 (Fig. 5d). The prominent role of eIF1 and eIF2 in holding eIF3c-NTD at the 40S interface is supported by biochemical data demonstrating the essential role of eIF2 for the antiassociation activity of eIF3, with eIF1 greatly enhancing this activity^21^. The release of eIF2–GDP from 48S-5 leads to nearly complete dissociation of the eIF3c-NTD from the 40S, paving the way for 60S joining to 48S in the presence of eIF3 (Fig. 5d,e).

A comparison with 48S structures from yeast^9,12^ suggests that this eIF3-mediated regulation of subunit joining may be evolutionarily conserved, despite the different topology of yeast eIF3c (refs. ^9,28^) (Extended Data Fig. 7a,d). The globular eIF3c-NTD binds to the same site on the 40S as in the human system. In yeast, however, eIF3c-NTT upstream of the NTD interacts with eIF1 (Extended Data Fig. 7d, left and center), taking over the role of the eIF3c linker region in the human system. In yeast system, eIF3c-NTD occupancy decreases upon replacement of eIF1 by eIF5 before GTP hydrolysis (Extended Data Fig. 7a, bottom and 7d, right)^9,12^, indicating a similar coupling between the 48S state and the antiassociation activity of eIF3. Additionally, three other eIF3 subunits (eIF3a/b/i) in yeast block eIF5B binding and premature 60S docking by shielding the intersubunit interface of the 40S (ref. ^9^). Notably, their antiassociation activity seems to be abolished already upon replacement of eIF1 by eIF5, indicating a different control mechanism by 48S remodeling^9,12^ (Extended Data Fig. 7d).

Recent in vivo studies revealed that eIF3 can persist on the 80S ribosome during elongation for up to 60 codons after initiation^22–25^. On the basis of the present 48S-5 structure, we generated models of eIF3 on the 80S ribosome, suggesting that the presence of eIF3 on the 80S ribosome is compatible with 60S joining and the major ribosomal rearrangements during translation elongation (Fig. 5e and Extended Data Fig. 7e,f). Notably, the models imply an extended contact surface between eIF3c-CTD and eL30 with substantial charge complementarity, which could contribute to stabilizing eIF3 on the 80S ribosome (Fig. 5e), in addition to the extensive mRNA–eIF3 interactions observed in the structure (Fig. 5c). Modeling of yeast 80S–eIF3 complexes further suggests a similar eIF3c-CTD–eL30 contact interface with complementary charges (Extended Data Fig. 7g), indicating a conserved mechanism for stabilizing eIF3 on the 80S ribosome.

## Discussion

**Fig. 6:**
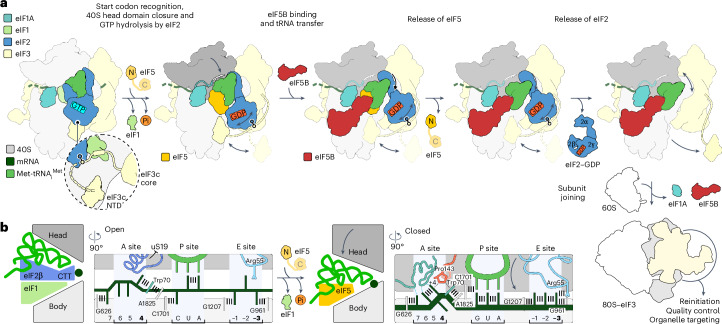
Molecular mechanism of 48S remodeling during translation initiation in human cells. **a**, Pathway of 48S remodeling. The open 48S with eIF1 bound scans the mRNA for start codons. Start codon recognition triggers closure of the 40S, replacement of eIF1 by eIF5 and, upon eIF5-induced GTP hydrolysis, remodeling of eIF2, leading to tRNA release from eIF2 and making way for eIF5B recruitment. eIF5B initially binds to the 48S in the presence of eIF2 and eIF5 and takes over the CCA end of Met-tRNA_i_^Met^. Sequential dissociation of eIF5 and eIF2 tunes the gradual positioning and dynamics of Met-tRNA_i_^Met^ for subunit joining. Close-up view shows that, in the open 48S, eIF3c-NTD is anchored to the 40S by its linker that interacts with eIF1 and eIF2, thereby blocking premature 60S docking. Release of eIF1 and then eIF2 results in almost complete dissociation of eIF3c-NTD, enabling eIF3 to stay on the 40S upon and beyond 60S joining and to function in the context of the 80S ribosome^22–25^ (80S–eIF3, bottom right). **b**, 40S head domain closure and the structural role of mRNA context flanking the start codon. Left: during mRNA scanning, eIF1 and eIF2β form a wedge that keeps the 48S in an open state, while mRNA residues at key −3 and +4 positions stabilize the mRNA for codon scanning by Met-tRNA_i_^Met^ in the P_out_ position. eIF2β keeps uS19 away from the A site. Right: start codon recognition by P_in_ Met-tRNA_i_^Met^ displaces eIF1 and eIF2β from the decoding center and induces 40S head closure, which is stabilized by interactions of uS19, eIF1A-NTT and eIF2α with the mRNA context in the posthydrolysis 48S. Notably, Kozak interactions in the A site induce a compact mRNA configuration, resulting in a tight mRNA–eIF1A–40S network. Key positions appear in bold.

Our structural analysis delineates the sequential remodeling events and visualizes the key commitment steps during translation initiation on the human 48S complex (Fig. 6 and Supplementary Video 2). We unravel the regulatory role of the Kozak sequence in both open and closed 48S states. In the open state, the Kozak sequence stabilizes the mRNA, ensuring accurate reading of the start codon by the tRNA during scanning. In the closed posthydrolysis state, the Kozak sequence, in concert with eIF1A-NTT and uS19, helps to form a compact mRNA structure within the A site and supports eIF2α in bridging the 40S head and body at the E site, resulting in extensive interaction networks that contribute to start site commitment by arresting the mRNA and stabilizing 40S closure. These findings provide a structural framework for understanding the intricate interplay of Kozak sequences, eIFs and ribosomal proteins in regulating translation^3,44,45^.

Furthermore, the study reveals the role of GTP hydrolysis by eIF2 in orchestrating late steps of translation initiation. The exchange of eIFs within the complex and structural rearrangements triggered by GTP hydrolysis lock the ribosome on the selected start site, initiating gradual repositioning of the initiator tRNA and retraction of eIF3 from the 40S subunit interface and paving the way for the joining of the 60S subunit. Lastly, our findings suggest a model for the human 80S ribosome-bound eIF3 complex. This model is in line with recent insights into the role of eIF3 in early translation elongation, including its involvement in reinitiation, membrane targeting and recruitment of protein quality control factors^22–25^, offering a more comprehensive view of translation initiation in human cells.

## Methods

### Preparation of human 48S complexes for cryo-EM

Native human 40S and eIFs (eIF2, eIF5B, eIF3 with eIF4G and eIF4E) were purified from HeLa cells using established protocols^10,50^, except for the omission of the final chromatography step to separate eIF3 and eIF4F. The quality of all preparations and the relative concentration of the eIF3 and eIF4F components in the eIF3 and eIF4F preparations were estimated by mass spectrometry. Recombinant human initiation factors (eIF1, eIF1A, eIF4A, eIF4B and eIF5), [^3^H]Met-tRNA_i_^Met^ and model mRNA were prepared as previously described^10^. The model mRNA features an unstructured 5′UTR, eliminating the need for 5′ capping and eIF4F recognition. This uncapped and unstructured mRNA is efficiently translated with the minimal set of translation factors^10,51,52^ and serves as a suitable model for studying start codon recognition without potential rate-limiting steps associated with eIF4-dependent cap recruitment or scanning. The functional activity of 48S and 80S complexes was confirmed through toe printing^10^. The 48S initiation complex induces a robust toe-printing stop on the cognate start codon (AUG), which is absent on the noncognate codon (CUC), whereas near-cognate codons (including AUC) allow the formation of a specific but labile complex^10^.

To prepare the human 48S initiation complex, 40S subunits (0.3–0.4 µM) were incubated with an excess of eIF1 (1.5 µM), eIF1A (1.5 µM), eIF2 (1 µM), eIF3, eIF4E, and eIF4G (1 µM eIF3, 0.4 µM eIF4G and 0.3 µM eIF4E; that is, keeping the major eIF4F components in about a 1:1 ratio to the 40S subunit), eIF4A (1.2 µM), eIF4B (0.75 µM), eIF5 (4 µM), eIF5B (0.8–1 µM), [^3^H]Met-tRNA_i_^Met^ (1.2 µM) and mRNA (1.5 µM) in reaction buffer (20 mM HEPES pH 7.5, 95 mM potassium acetate, 3.75 mM magnesium acetate, 1 mM ATP, 0.5 mM GTP, 2 mM DTT and 0.25 mM spermidine) for 5, 10 or 15 min at 37 °C. Then, complexes were incubated with the crosslinking reagent bis(sulfosuccinimidyl)suberate (BS3; Sigma Aldrich) (2 mM) for 10 min at room temperature to stabilize complexes for cryo-EM preparation. Sucrose and glycerol in the reaction mixtures were removed by Zeba Spin columns (Thermo Fisher Scientific), preincubated with 0.1 mg ml^−1^ gelatin to improve sample recovery^10^.

### Cryo-EM analysis

Cryo-EM grids were prepared by applying 4.5 µl of the 48S reaction mixture onto glow-discharged UltraAuFoil R2.5/1.5 gold grids (Quantifoil company, Jena) or glow-discharged R3.5/1 copper grids (Quantifoil company, Jena) covered with a freshly floated, continuous thin carbon film. Grids were vitrified using a custom-made manual plunge-freezer in a cold room at 4 °C and 90% relative humidity, blotting the gold grids for 8–10 s with Whatman no. 1 filter paper from the back and the carbon-covered grids with prewetted filter paper for about 8 s from the front.

Cryo-EM data acquisition was performed on a Titan Krios G1 microscope at 300 kV acceleration voltage with a Falcon III direct electron detector and XFEG electron source (Thermo Fisher Scientific) using a C_S_-corrector (CEOS) aligned with the CETCORPLUS 4.6.9 software package (CEOS) for aberration correction. Cryo-EM movie images at 4,096 × 4,096 pixels were collected in integration mode with the EPU 2.3 software (Thermo Fisher Scientific) to obtain 20 frames for each 1–2-s movie at a defocus range of 0.2–2.5 μm, a total dose of ~40–50 electrons per Å^2^ and a nominal magnification of ×59,000, corresponding to a final pixel size of 1.16 Å per pixel.

For cryo-EM image processing, all cryo-EM data were combined and processed in RELION-4.0 (ref. ^53^) (Extended Data Figs. 2 and 3), unless indicated otherwise. Cryo-EM movie frames were averaged using global motion correction and dose weighting, parameters for the contrast transfer function (CTF) were estimated with GCTF 1.0.6 (ref. ^54^) and particles were selected in Gautomatch 0.56 (K. Zhang, MRC-LMB, Cambridge) using a vacant 40S as template. The selected particle images were extracted at fourfold binned pixel sizes for two-dimensional (2D) classification to sort for particle quality, followed by three-dimensional (3D) classifications to sort for particle quality, global 40S conformation and eIF presence, yielding two major particle populations of 48S complexes in the open and closed states. The two particle populations were processed separately in the subsequent steps, using similar strategies to those outlined in Extended Data Fig. 2b. In brief, particle images were re-extracted at the final, unbinned pixel size of 1.16 Å per pixel in a 360 × 360 box, 3D refinement was performed for refinement of CTF parameters (scaling, coma and higher-order aberrations) and per-particle motion correction was performed using Bayesian polishing^55,56^. The particles were then extensively sorted by multiple steps of focused classification with signal subtraction (FCwSS) for the presence and definition of individual eIFs and Met-tRNA_i_^Met^; eIF4A/B and eIF4F were not clearly visible in the complexes. We obtained five 48S structures with distinct eIF compositions determined by gold-standard refinement at overall resolutions of 2.9 to 3.7 Å (Extended Data Fig. 1a–c). In addition, we determined the structure of a late-stage 48S with Met-tRNA^Met^, eIF1A and eIF3, after eIF2 release, but without eIF5B at 3.3 Å resolution (Extended Data Fig. 2b,d).

While the extensive sorting for eIF presence yielded high occupancy with specific eIFs in each state, conformational dynamics of individual eIFs were partly still substantial (Extended Data Figs. 1a and 2c). These local dynamics were resolved (1) by focused refinement for the octameric eIF3 core, followed by 3D flexible refinement in cryoSparc 4.4.0 (3DFlex)^57,58^, and (2) by additional FCwSS, resulting in substates for the eIF3b/i subcomplex and for eIF2, Met-tRNA_i_^Met^ and/or eIF5B (Extended Data Figs. 1d–f, 2b,c and 3a–c). The 3DFlex refinement of the eIF3 core in the open state generally improved density as compared to focused refinement, in terms of both nominal resolution and map quality, and the 3DFlex-based map was used for atomic model refinement of the entire eIF3 core in the open state (Extended Data Fig. 3b). For the eIF3 core in the closed state, 3DFlex resulted in slight overall improvements and Fourier shell resolution curves computed from unmasked half-maps, but not from the tightly masked half-maps, showed spurious correlations at high resolutions, suggesting overfitting of background noise at <4 Å resolution. However, local resolution and map quality for the k/l subunits of eIF3 were much improved in the 3DFlex-refined density, resolving these highly flexible subunits at about 6 Å resolution (that is, at resolutions not affected by the high-resolution overfitting) (Extended Data Fig. 1e). Consequently, the 3DFlex map was used for atomic model building of eIF3 k/l subunits, whereas atomic model refinement of the full eIF3 core in the closed state was based on the map from focused refinement. To quantify binding of the eIF3c-NTD to the 40S, FCwSS was performed focusing on the NTD’s binding site for each of the final particle populations (48S-1 to 48S-5; Extended Data Fig. 3d).

### Atomic model refinement

Initial atomic models were assembled and rigidly fit into the respective cryo-EM maps using ChimeraX 1.4 (ref. ^59^). For the subsequent manual refinements in Coot 0.9.8.1 (ref. ^60^), cryo-EM maps were low-pass filtered to the respective local resolution of the area of interest, as detailed below. The assembled models were initially refined into the maps using standard geometric restraints and distance restraints with a general 4.3 Å cutoff. For subsequent rounds of manual refinement, distance restraints were only used for structural elements resolved at resolutions below 5 Å, with cutoffs ranging from 3.7 to 5 Å dependent on the local map quality, while base-pairing restraints were used for the tRNA; distance restraints were created per chain and locally for contact areas. Final atomic models were real-space refined into full maps in Phenix^61^ over five macrocycles of 300 iterations each, using reference model, secondary structure and metal coordination restraints. Geometry outliers in the resulting structures were first corrected manually in Coot, followed by another Phenix real-space refinement over three macrocycles of 100 iterations each (Table 1).

#### eIF3

The octameric core of eIF3 in the closed state was manually modeled into the 3.5 Å map obtained by focused refinement of all closed-state particles using the corresponding chains of the human 48S scanning complex (Protein Data Bank (PDB) 6ZMW)^7^, except for the highly dynamic eIF3k and eIF3l subunits. The latter subunits were manually modeled into the 3DFlex map of the closed state, which was low-pass filtered to 7 Å resolution, using the initial model for eIF3k from PDB 6ZMW (ref. ^7^) and for eIF3l from an AlphaFold prediction^62,63^ (AF-Q9Y262-F1). This allowed us to expand eIF3l structure by 137 residues toward the N terminus. The overall model of the eIF3 core in the closed state was then refined using Phenix into the individual closed state maps (48S-2 to 48S-5) and into the 3.8 Å 3DFlex map of the eIF3 core in the open state. The latter model was then refined into the full 48S-1 map. The eIF3b/i subunits with ES6 were manually refined into the density maps of individual eIF3b/i substates, low-pass filtered to 15–20 Å resolution, using the corresponding chains from PDB 6ZMW (ref. ^7^) as starting models. The resulting models for the eIF3b/i major substate in open and closed 48S were then refined with Phenix into the full 48S-1 map and 48S-2 to 48S-5 maps, respectively.

#### 48S-5

The 48S-5 state at 2.9 Å resolution was modeled first to obtain improved models of the 40S, eIF1A and eIF5B, which then served to model other structures. The 40S and eIF1A were manually refined on the basis of PDB 7QP7 (ref. ^10^), the modification m^1^acp^3^Ψ1248 of 18S rRNA was added using Coot 0.9.8.1, geometric structure restraints were created using the Grade Web Server (http://grade.globalphasing.org/) and the preparation of structural restraints for modified 18S rRNA residues was performed with the phenix.ready set in Phenix. Ribosomal proteins eS19 and eS13 could be manually modeled to their C termini and the eIF1A-NTT could be modeled to Pro2. An initial model of eIF5B was taken from an AlphaFold prediction^62,63^ (AF-O60841-F1) removing the first 600 residues, which are predicted to be unstructured and were not resolved in the present or previous structures. The catalytic pocket of eIF5B with GTP was modeled on the basis of PDB 4NCN (ref. ^64^) and the model for Met-tRNA_i_^Met^ was taken from 48S-1 (as described below), while the interface between the tRNA’s CCA end and eIF5B was modeled on the basis of the yeast 80S complex with eIF5B and Met-tRNA_i_^Met^ (PDB 6WOO)^65^. The substates of the eIF1A–eIF5B-CTD–tRNA complex (48S-5-1 to 48S-5-3) were refined manually in Coot at 3.1–3.7 Å resolution using local and chain refinements.

#### 48S-1

eIF1A and eIF2α were modeled on the basis of human closed 48S (PDB 7QP7)^10^, while Met-tRNA_i_^Met^, eIF1 and eIF2β were modeled on the basis of the human 43S preinitiation complex (PDB 7A09)^28^. We could model the structure of eIF2β to its C terminus in the overall map at 3.4 Å resolution, adding the Zn cluster on the basis of the structure of aIF2 (PDB 1KK1)^34^. eIF2γ was modeled into the 48S-1 map low-pass filtered to 3.75–5 Å resolution, using an AlphaFold prediction^62,63^ (AF-P41091-F1) and adding GTP-Mg on the basis of the aIF2–GDPNP structure (PDB 1KK1)^34^. Notably, we could also manually trace the backbone of the NTT of eIF3c at 6 Å resolution to about residue 34, showing that the human eIF3c-NTT reaches back to the decoding center.

#### 48S-2 to 48S-4

The NTD of eIF5 in 48S-2 and 48S-3 was taken from AlphaFold^62,63^ (AF-P55010-F1). To model the low-resolution substates of the highly dynamic eIF2–GDP, models for eIF2α and eIF2γ were taken from 48S-1 and the switch 1 region of eIF2γ was truncated to reflect its disordering upon GTP hydrolysis^34^. The eIF2 subunits were then rigidly fit and refined into the cryo-EM densities of the substates at 12–20 Å resolution, using additionally generated distance restraints with a 5 Å cutoff.

#### 48S without eIF5B

The initial model was based on 48S-5, removing eIF5B and the very flexible CCA end of the tRNA; the tRNA model was refined into the map low-pass filtered to 12–20 Å resolution.

### Principal component analysis of tRNA motions

To follow the tRNA trajectory from 48S-1 to 48S-5 (Fig. 4b), a principal component analysis was performed with a custom script written in Python 3.8, using package scikit-learn 1.1.3 (ref. ^66^). The following atoms were used for the PCA: P of C56 for the tRNA elbow and P of A73 for the tRNA CCA end. Plots were obtained using Matplotlib 3.5.3 in Python 3.8.

### 3D-printed models for simulating ribosomal subunit joining

Volumes for 3D printing were rendered at 5 Å resolution using the command molmap in ChimeraX 1.4 on the basis of the atomic models of 48S-5, the 48S without eIF5B and the 60S (PDB 6QZP)^49^. The 3D models were then printed at the same scale on an Agilista-3200W printer (Keyence) with software Modeling Studio 1.8.0.0 (Keyence) using the flexible AR-G1H material with AR-S1 support material (Keyence). Manual docking of the 3D-printed model of the respective 48S model on the 60S indicated no clashes between 48S-bound eIFs or Met-tRNA_i_^Met^ and the 60S upon subunit joining (Supplementary Video 1); the resulting 3D models of the 80S ribosome measured a maximum of ~90 mm in diameter.

### Reporting summary

Further information on research design is available in the Nature Portfolio Reporting Summary linked to this article.

## Online content

Any methods, additional references, Nature Portfolio reporting summaries, source data, extended data, supplementary information, acknowledgements, peer review information; details of author contributions and competing interests; and statements of data and code availability are available at 10.1038/s41594-024-01378-4.

## Supplementary information

Reporting Summary

Supplementary Video 1 Simulation of ribosomal subunit joining with 3D-printed models. The video illustrates the compatibility of the present late-stage eIF3-bound 48S structures with joining of the large ribosomal subunit, as observed by docking of flexible 3D-printed models.

Supplementary Video 2 Structural mechanism of human 48S remodeling. The animation shows the mechanism of human translation initiation from codon scanning toward subunit joining. The animation is based on the experimental structures; transitions between structures were rendered by morphing.

## Data Availability

Cryo-EM maps and associated coordinates of atomic models were deposited to the EMDB and PDB with the following accession codes: EMD-17696 and PDB 8PJ1 (48S-1), EMD-17697 and PDB 8PJ2 (48S-2), EMD-17698 and PDB 8PJ3 (48S-3), EMD-17699 and PDB 8PJ4 (48S-4), EMD-17700 and PDB 8PJ5 (48S-5), EMD-17701 and PDB 8PJ6 (off-pathway 48S without eIF5B) and EMD-19128 and PDB 8RG0 (eIF3 core in closed 48S). Cryo-EM micrographs and particle images were deposited to the Electron Microscopy Public Image Archive (EMPIAR) with accession code EMPIAR-12094. In addition, the following structures were used in the present study and can be retrieved from the PDB, AlphaFold Protein Structure Database and EMDB: PDB 6QZP, PDB 6ZMW, PDB 7QP7, PDB 4NCN, PDB 6WOO, PDB 7A09, PDB 1KK1, PDB 1KK2, PDB 6YAL, PDB 6FYX, PDB 3JAG, PDB 7TQL, PDB 6OM0, PDB 6FYY, AF-Q9Y262-F1, AF-O60841-F1, AF-P41091-F1, AF-P55010-F1, EMD-0057, EMD-0058 and EMD-4330. All other data supporting the findings of this study are available within the paper and the Supplementary Information.

## Code availability

The script for principal component analysis of tRNA motions is available on GitHub (https://github.com/Molecular-Machines-in-Motion/tRNA_PCA).

## References

[1] Jackson, R. J., Hellen, C. U. & Pestova, T. V. The mechanism of eukaryotic translation initiation and principles of its regulation. *Nat. Rev. Mol. Cell Biol.* **11**, 113–127 (2010). 20094052 10.1038/nrm2838 PMC4461372

[2] Hinnebusch, A. G. Structural insights into the mechanism of scanning and start codon recognition in eukaryotic translation initiation. *Trends Biochem. Sci.* **42**, 589–611 (2017). 28442192 10.1016/j.tibs.2017.03.004

[3] Merrick, W. C. & Pavitt, G. D. Protein synthesis initiation in eukaryotic cells. *Cold Spring Harb. Perspect. Biol.* **10**, a033092 (2018). 29735639 10.1101/cshperspect.a033092 PMC6280705

[4] Hashem, Y. & Frank, J. The jigsaw puzzle of mRNA translation initiation in eukaryotes: a decade of structures unraveling the mechanics of the process. *Annu Rev. Biophys.* **47**, 125–151 (2018). 29494255 10.1146/annurev-biophys-070816-034034 PMC6318078

[5] Sokabe, M. & Fraser, C. S. Toward a kinetic understanding of eukaryotic translation. *Cold Spring Harb. Perspect. Biol.* **11**, a032706 (2019). 29959192 10.1101/cshperspect.a032706 PMC6360857

[6] Paulin, F. E., Campbell, L. E., O’Brien, K., Loughlin, J. & Proud, C. G. Eukaryotic translation initiation factor 5 (eIF5) acts as a classical GTPase-activator protein. *Curr. Biol.* **11**, 55–59 (2001). 11166181 10.1016/s0960-9822(00)00025-7

[7] Brito Querido, J. et al. Structure of a human 48S translational initiation complex. *Science* **369**, 1220–1227 (2020). 32883864 10.1126/science.aba4904 PMC7116333

[8] Llacer, J. L. et al. Conformational differences between open and closed states of the eukaryotic translation initiation complex. *Mol. Cell* **59**, 399–412 (2015). 26212456 10.1016/j.molcel.2015.06.033 PMC4534855

[9] Llacer, J. L. et al. Large-scale movement of eIF3 domains during translation initiation modulate start codon selection. *Nucleic Acids Res.* **49**, 11491–11511 (2021). 34648019 10.1093/nar/gkab908 PMC8599844

[10] Yi, S. H. et al. Conformational rearrangements upon start codon recognition in human 48S translation initiation complex. *Nucleic Acids Res.* **50**, 5282–5298 (2022). 35489072 10.1093/nar/gkac283 PMC9122606

[11] Simonetti, A., Guca, E., Bochler, A., Kuhn, L. & Hashem, Y. Structural insights into the mammalian late-stage initiation complexes. *Cell Rep.* **31**, 107497 (2020). 32268096 10.1016/j.celrep.2020.03.061 PMC7166083

[12] Llacer, J. L. et al. Translational initiation factor eIF5 replaces eIF1 on the 40S ribosomal subunit to promote start-codon recognition. *eLife* **7**, e39273 (2018). 30475211 10.7554/eLife.39273 PMC6298780

[13] Brito Querido, J. et al. The structure of a human translation initiation complex reveals two independent roles for the helicase eIF4A. *Nat. Struct. Mol. Biol.* **31**, 455–464 (2024). 38287194 10.1038/s41594-023-01196-0 PMC10948362

[14] Unbehaun, A., Borukhov, S. I., Hellen, C. U. & Pestova, T. V. Release of initiation factors from 48S complexes during ribosomal subunit joining and the link between establishment of codon–anticodon base-pairing and hydrolysis of eIF2-bound GTP. *Genes Dev.* **18**, 3078–3093 (2004). 15601822 10.1101/gad.1255704 PMC535918

[15] Algire, M. A., Maag, D. & Lorsch, J. R. Pi release from eIF2, not GTP hydrolysis, is the step controlled by start-site selection during eukaryotic translation initiation. *Mol. Cell* **20**, 251–262 (2005). 16246727 10.1016/j.molcel.2005.09.008

[16] Pisarev, A. V. et al. Specific functional interactions of nucleotides at key −3 and +4 positions flanking the initiation codon with components of the mammalian 48S translation initiation complex. *Genes Dev.* **20**, 624–636 (2006). 16510876 10.1101/gad.1397906 PMC1410799

[17] Pestova, T. V. et al. The joining of ribosomal subunits in eukaryotes requires eIF5B. *Nature* **403**, 332–335 (2000). 10659855 10.1038/35002118

[18] Lapointe, C. P. et al. eIF5B and eIF1A reorient initiator tRNA to allow ribosomal subunit joining. *Nature* **607**, 185–190 (2022). 35732735 10.1038/s41586-022-04858-z PMC9728550

[19] Kozak, M. Structural features in eukaryotic mRNAs that modulate the initiation of translation. *J. Biol. Chem.* **266**, 19867–19870 (1991). 1939050

[20] Benitez-Cantos, M. S. et al. Translation initiation downstream from annotated start codons in human mRNAs coevolves with the Kozak context. *Genome Res.* **30**, 974–984 (2020). 32669370 10.1101/gr.257352.119 PMC7397870

[21] Kolupaeva, V. G., Unbehaun, A., Lomakin, I. B., Hellen, C. U. & Pestova, T. V. Binding of eukaryotic initiation factor 3 to ribosomal 40S subunits and its role in ribosomal dissociation and anti-association. *RNA* **11**, 470–486 (2005). 15703437 10.1261/rna.7215305 PMC1370736

[22] Wagner, S. et al. Selective translation complex profiling reveals staged initiation and co-translational assembly of initiation factor complexes. *Mol. Cell* **79**, 546–560 (2020). 32589964 10.1016/j.molcel.2020.06.004 PMC7447980

[23] Lin, Y. et al. eIF3 associates with 80S ribosomes to promote translation elongation, mitochondrial homeostasis, and muscle health. *Mol. Cell* **79**, 575–587 (2020). 32589965 10.1016/j.molcel.2020.06.003

[24] Bohlen, J., Fenzl, K., Kramer, G., Bukau, B. & Teleman, A. A. Selective 40S footprinting reveals cap-tethered ribosome scanning in human cells. *Mol. Cell* **79**, 561–574 (2020). 32589966 10.1016/j.molcel.2020.06.005

[25] Mohammad, M. P., Pondelickova, V. M., Zeman, J., Gunisova, S. & Valasek, L. S. In vivo evidence that eIF3 stays bound to ribosomes elongating and terminating on short upstream ORFs to promote reinitiation. *Nucleic Acids Res.* **45**, 2658–2674 (2017). 28119417 10.1093/nar/gkx049 PMC5389480

[26] Aylett, C. H., Boehringer, D., Erzberger, J. P., Schaefer, T. & Ban, N. Structure of a yeast 40S–eIF1–eIF1A–eIF3–eIF3j initiation complex. *Nat. Struct. Mol. Biol.* **22**, 269–271 (2015). 25664723 10.1038/nsmb.2963

[27] Sokabe, M. & Fraser, C. S. A helicase-independent activity of eIF4A in promoting mRNA recruitment to the human ribosome. *Proc. Natl Acad. Sci. USA* **114**, 6304–6309 (2017). 28559306 10.1073/pnas.1620426114 PMC5474785

[28] Kratzat, H. et al. A structural inventory of native ribosomal ABCE1–43S pre-initiation complexes. *EMBO J.* **40**, e105179 (2021). 33289941 10.15252/embj.2020105179 PMC7780240

[29] Wang, J. et al. Rapid 40S scanning and its regulation by mRNA structure during eukaryotic translation initiation. *Cell* **185**, 4474–4487 (2022). 36334590 10.1016/j.cell.2022.10.005 PMC9691599

[30] Mitchell, S. F. & Lorsch, J. R. Should I stay or should I go? Eukaryotic translation initiation factors 1 and 1A control start codon recognition. *J. Biol. Chem.* **283**, 27345–27349 (2008). 18593708 10.1074/jbc.R800031200 PMC2562056

[31] Thakur, A., Marler, L. & Hinnebusch, A. G. A network of eIF2β interactions with eIF1 and Met-tRNA_i_ promotes accurate start codon selection by the translation preinitiation complex. *Nucleic Acids Res.* **47**, 2574–2593 (2019). 30576497 10.1093/nar/gky1274 PMC6411837

[32] Rabl, J., Leibundgut, M., Ataide, S. F., Haag, A. & Ban, N. Crystal structure of the eukaryotic 40S ribosomal subunit in complex with initiation factor 1. *Science* **331**, 730–736 (2011). 21205638 10.1126/science.1198308

[33] Babaian, A. et al. Loss of m^1^acp^3^ψ ribosomal RNA modification is a major feature of cancer. *Cell Rep.* **31**, 107611 (2020). 32375039 10.1016/j.celrep.2020.107611

[34] Schmitt, E., Blanquet, S. & Mechulam, Y. The large subunit of initiation factor aIF2 is a close structural homologue of elongation factors. *EMBO J.* **21**, 1821–1832 (2002). 11927566 10.1093/emboj/21.7.1821 PMC125960

[35] Kapp, L. D. & Lorsch, J. R. GTP-dependent recognition of the methionine moiety on initiator tRNA by translation factor eIF2. *J. Mol. Biol.* **335**, 923–936 (2004). 14698289 10.1016/j.jmb.2003.11.025

[36] Fekete, C. A. et al. N- and C-terminal residues of eIF1A have opposing effects on the fidelity of start codon selection. *EMBO J.* **26**, 1602–1614 (2007). 17332751 10.1038/sj.emboj.7601613 PMC1829380

[37] Martin, M. et al. Exome sequencing identifies recurrent somatic mutations in *EIF1AX* and *SF3B1* in uveal melanoma with disomy 3. *Nat. Genet.* **45**, 933–936 (2013). 23793026 10.1038/ng.2674 PMC4307600

[38] Hamilton, R., Watanabe, C. K. & de Boer, H. A. Compilation and comparison of the sequence context around the AUG startcodons in *Saccharomyces cerevisiae* mRNAs. *Nucleic Acids Res.* **15**, 3581–3593 (1987). 3554144 10.1093/nar/15.8.3581 PMC340751

[39] Brown, A., Shao, S., Murray, J., Hegde, R. S. & Ramakrishnan, V. Structural basis for stop codon recognition in eukaryotes. *Nature* **524**, 493–496 (2015). 26245381 10.1038/nature14896 PMC4591471

[40] Matheisl, S., Berninghausen, O., Becker, T. & Beckmann, R. Structure of a human translation termination complex. *Nucleic Acids Res.* **43**, 8615–8626 (2015). 26384426 10.1093/nar/gkv909 PMC4605324

[41] Lin, K. Y., Nag, N., Pestova, T. V. & Marintchev, A. Human eIF5 and eIF1A compete for binding to eIF5B. *Biochemistry* **57**, 5910–5920 (2018). 30211544 10.1021/acs.biochem.8b00839 PMC6177315

[42] Guillon, L., Schmitt, E., Blanquet, S. & Mechulam, Y. Initiator tRNA binding by e/aIF5B, the eukaryotic/archaeal homologue of bacterial initiation factor IF2. *Biochemistry* **44**, 15594–15601 (2005). 16300409 10.1021/bi051514j

[43] Bochler, A. et al. Structural differences in translation initiation between pathogenic trypanosomatids and their mammalian hosts. *Cell Rep.* **33**, 108534 (2020). 33357443 10.1016/j.celrep.2020.108534 PMC7773551

[44] Hinnebusch, A. G., Ivanov, I. P. & Sonenberg, N. Translational control by 5′-untranslated regions of eukaryotic mRNAs. *Science* **352**, 1413–1416 (2016). 27313038 10.1126/science.aad9868 PMC7422601

[45] Hernandez, G., Osnaya, V. G. & Perez-Martinez, X. Conservation and variability of the AUG initiation codon context in eukaryotes. *Trends Biochem. Sci.* **44**, 1009–1021 (2019). 31353284 10.1016/j.tibs.2019.07.001

[46] Das, S. & Maitra, U. Mutational analysis of mammalian translation initiation factor 5 (eIF5): role of interaction between the beta subunit of eIF2 and eIF5 in eIF5 function in vitro and in vivo. *Mol. Cell Biol.* **20**, 3942–3950 (2000). 10805737 10.1128/mcb.20.11.3942-3950.2000 PMC85746

[47] Asano, K. et al. Multiple roles for the C-terminal domain of eIF5 in translation initiation complex assembly and GTPase activation. *EMBO J.* **20**, 2326–2337 (2001). 11331597 10.1093/emboj/20.9.2326 PMC125443

[48] Singh, C. R. et al. An eIF5/eIF2 complex antagonizes guanine nucleotide exchange by eIF2B during translation initiation. *EMBO J.* **25**, 4537–4546 (2006). 16990799 10.1038/sj.emboj.7601339 PMC1589998

[49] Natchiar, S. K., Myasnikov, A. G., Kratzat, H., Hazemann, I. & Klaholz, B. P. Visualization of chemical modifications in the human 80S ribosome structure. *Nature* **551**, 472–477 (2017). 29143818 10.1038/nature24482

[50] Pisarev, A. V., Unbehaun, A., Hellen, C. U. & Pestova, T. V. Assembly and analysis of eukaryotic translation initiation complexes. *Methods Enzymol.* **430**, 147–177 (2007). 17913638 10.1016/S0076-6879(07)30007-4

[51] Kumar, P., Hellen, C. U. & Pestova, T. V. Toward the mechanism of eIF4F-mediated ribosomal attachment to mammalian capped mRNAs. *Genes Dev.* **30**, 1573–1588 (2016). 27401559 10.1101/gad.282418.116 PMC4949329

[52] Pestova, T. V. & Kolupaeva, V. G. The roles of individual eukaryotic translation initiation factors in ribosomal scanning and initiation codon selection. *Genes Dev.* **16**, 2906–2922 (2002). 12435632 10.1101/gad.1020902 PMC187480

[53] Kimanius, D., Dong, L., Sharov, G., Nakane, T. & Scheres, S. H. W. New tools for automated cryo-EM single-particle analysis in RELION-4.0. *Biochem. J.* **478**, 4169–4185 (2021). 34783343 10.1042/BCJ20210708 PMC8786306

[54] Zhang, K. Gctf: real-time CTF determination and correction. *J. Struct. Biol.* **193**, 1–12 (2016). 26592709 10.1016/j.jsb.2015.11.003 PMC4711343

[55] Zivanov, J., Nakane, T. & Scheres, S. H. W. A Bayesian approach to beam-induced motion correction in cryo-EM single-particle analysis. *IUCrJ* **6**, 5–17 (2019). 30713699 10.1107/S205225251801463X PMC6327179

[56] Zivanov, J., Nakane, T. & Scheres, S. H. W. Estimation of high-order aberrations and anisotropic magnification from cryo-EM data sets in RELION-3.1. *IUCrJ* **7**, 253–267 (2020). 32148853 10.1107/S2052252520000081 PMC7055373

[57] Punjani, A., Rubinstein, J. L., Fleet, D. J. & Brubaker, M. A. cryoSPARC: algorithms for rapid unsupervised cryo-EM structure determination. *Nat. Methods* **14**, 290–296 (2017). 28165473 10.1038/nmeth.4169

[58] Punjani, A. & Fleet, D. J. 3DFlex: determining structure and motion of flexible proteins from cryo-EM. *Nat. Methods* **20**, 860–870 (2023). 37169929 10.1038/s41592-023-01853-8 PMC10250194

[59] Pettersen, E. F. et al. UCSF ChimeraX: structure visualization for researchers, educators, and developers. *Protein Sci.* **30**, 70–82 (2021). 32881101 10.1002/pro.3943 PMC7737788

[60] Casanal, A., Lohkamp, B. & Emsley, P. Current developments in Coot for macromolecular model building of electron cryo-microscopy and crystallographic data. *Protein Sci.* **29**, 1069–1078 (2020). 31730249 10.1002/pro.3791 PMC7096722

[61] Liebschner, D. et al. Macromolecular structure determination using X-rays, neutrons and electrons: recent developments in Phenix. *Acta Crystallogr. D Struct. Biol.* **75**, 861–877 (2019). 31588918 10.1107/S2059798319011471 PMC6778852

[62] Jumper, J. et al. Highly accurate protein structure prediction with AlphaFold. *Nature* **596**, 583–589 (2021). 34265844 10.1038/s41586-021-03819-2 PMC8371605

[63] Varadi, M. et al. AlphaFold Protein Structure Database in 2024: providing structure coverage for over 214 million protein sequences. *Nucleic Acids Res.* **52**, D368–D375 (2024). 37933859 10.1093/nar/gkad1011 PMC10767828

[64] Kuhle, B. & Ficner, R. eIF5B employs a novel domain release mechanism to catalyze ribosomal subunit joining. *EMBO J.* **33**, 1177–1191 (2014). 24686316 10.1002/embj.201387344 PMC4193923

[65] Wang, J. et al. Structural basis for the transition from translation initiation to elongation by an 80S–eIF5B complex. *Nat. Commun.* **11**, 5003 (2020). 33024099 10.1038/s41467-020-18829-3 PMC7538418

[66] Pedregosa, F. et al. Scikit-learn: machine learning in Python. *J. Mach. Learn. Res.* **12**, 2825–2830 (2011).

[67] Li, W. et al. Structural basis for selective stalling of human ribosome nascent chain complexes by a drug-like molecule. *Nat. Struct. Mol. Biol.* **26**, 501–509 (2019). 31160784 10.1038/s41594-019-0236-8 PMC6919564

