# Author Correction: Structural basis for translational control by the human 48S initiation complex

**DOI:** 10.1038/s41594-026-01777-9

**Published:** 2026-02-26

**Authors:** Valentyn Petrychenko, Sung-Hui Yi, David Liedtke, Bee-Zen Peng, Marina V. Rodnina, Niels Fischer

**Affiliations:** 1 Project Group Molecular Machines in Motion, Department of Physical Biochemistry, Max Planck Institute for Multidisciplinary Sciences, Göttingen, Germany; 2 Department of Physical Biochemistry, Max Planck Institute for Multidisciplinary Sciences, Göttingen, Germany; 3 Present Address: Insempra GmbH, Planegg, Germany

**Keywords:** Cryoelectron microscopy, Ribosome

Correction to: *Nature Structural & Molecular Biology* 10.1038/s41594-024-01378-4, published online 17 September 2024.

In the originally published version of this article, residue numbering of eIF2α was incorrect because its sequence numbering in the initial atomic models erroneously started at zero, resulting in all eIF2α residues being offset by −1. Consequently, eIF2α residue labels in Fig. 3a,b were incorrect, and an arginine residue of eIF2α was incorrectly labelled as Arg54 in Figs. 3d and 6b, in Extended Data Fig. 5c,f, and at two positions in the text. The correct residue number is Arg55. These errors have now been corrected in the HTML and PDF versions of the article and in the corresponding atomic models deposited under PDB accession codes 8PJ1, 8PJ2, 8PJ3 and 8PJ4 (PDB entry versions 2.0 and higher).

